# GWAS-identified bipolar disorder risk allele in the *FADS1*/*2* gene region links mood episodes and unsaturated fatty acid metabolism in mutant mice

**DOI:** 10.1038/s41380-023-01988-2

**Published:** 2023-02-21

**Authors:** Hirona Yamamoto, Hyeon-Cheol Lee-Okada, Masashi Ikeda, Takumi Nakamura, Takeo Saito, Atsushi Takata, Takehiko Yokomizo, Nakao Iwata, Tadafumi Kato, Takaoki Kasahara

**Affiliations:** 1 Laboratory for Molecular Dynamics of Mental Disorders, RIKEN Center for Brain Science, Saitama, Japan; 2 Laboratory for Molecular Pathology of Psychiatric Disorders, RIKEN Center for Brain Science, Saitama, Japan; 3 Department of Neuropsychiatry, Graduate School of Medicine, The University of Tokyo, Tokyo, Japan; 4 Department of Biochemistry, Juntendo University School of Medicine, Tokyo, Japan; 5 Department of Psychiatry, Fujita Health University School of Medicine, Aichi, Japan; 6 Department of Psychiatry and Behavioral Science, Juntendo University Graduate School of Medicine, Tokyo, Japan; 7 Research Institute for Disease of Old Age, Juntendo University School of Medicine, Tokyo, Japan; 8 Career Development Program, RIKEN Center for Brain Science, Saitama, Japan; 9 Neurodegenerative Disorders Collaboration Laboratory, RIKEN Center for Brain Science, Saitama, Japan; 10 Present Address: Institute of Biology and Environmental Sciences, Carl von Ossietzky University of Oldenburg, Oldenburg, Germany

**Keywords:** Bipolar disorder, Neuroscience

## Abstract

Large-scale genome-wide association studies (GWASs) on bipolar disorder (BD) have implicated the involvement of the fatty acid desaturase (*FADS*) locus. These enzymes (FADS1 and FADS2) are involved in the metabolism of eicosapentaenoic acid (EPA) and docosahexaenoic acid (DHA), which are thought to potentially benefit patients with mood disorders. To model reductions in the activity of FADS1/2 affected by the susceptibility alleles, we generated mutant mice heterozygously lacking both *Fads1*/*2* genes. We measured wheel-running activity over six months and observed bipolar swings in activity, including hyperactivity and hypoactivity. The hyperactivity episodes, in which activity was far above the norm, usually lasted half a day; mice manifested significantly shorter immobility times on the behavioral despair test performed during these episodes. The hypoactivity episodes, which lasted for several weeks, were accompanied by abnormal circadian rhythms and a marked decrease in wheel running, a spontaneous behavior associated with motivation and reward systems. We comprehensively examined lipid composition in the brain and found that levels of certain lipids were significantly altered between wild-type and the heterozygous mutant mice, but no changes were consistent with both sexes and either DHA or EPA was not altered. However, supplementation with DHA or a mixture of DHA and EPA prevented these episodic behavioral changes. Here we propose that heterozygous *Fads1*/*2* knockout mice are a model of BD with robust constitutive, face, and predictive validity, as administration of the mood stabilizer lithium was also effective. This GWAS-based model helps to clarify how lipids and their metabolisms are involved in the pathogenesis and treatment of BD.

## Introduction

Bipolar disorder (BD) is a chronic mental illness characterized by recurrent manic and depressive episodes interspersed with an absence of symptoms (a euthymic state). Large-scale genome-wide association studies (GWASs) have identified dozens of loci associated with BD [1–3]. Among them, the *FADS1*/*2* region was first highlighted in Japanese population [1] and replicated in the large European population [2, 3], being the only locus with a genome-wide significant difference in multiple populations.

**Fig. 1:**
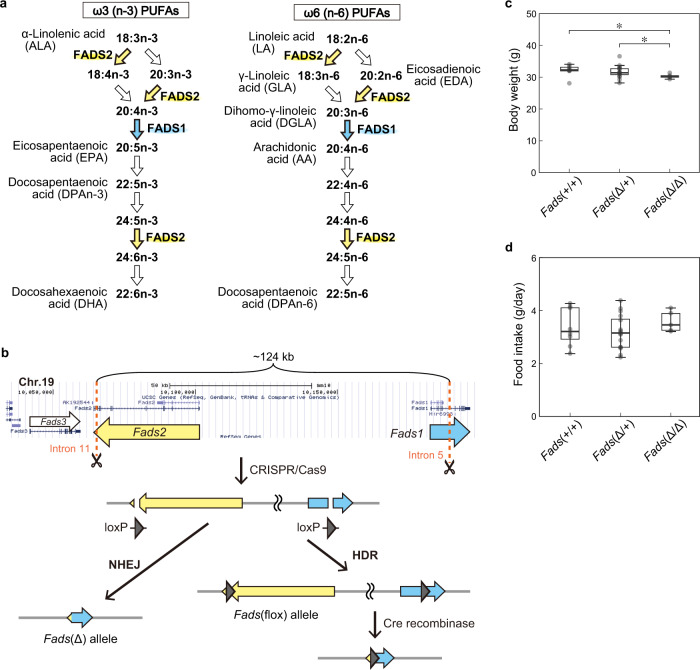
Generation of mutant mice. **a** The PUFA biosynthesis pathways. The desaturation reactions of ω3 and ω6 PUFAs are catalyzed by FADS1 (cyan arrows) or FADS2 (yellow arrows). **b** A strategy for simultaneous generation of alleles using the CRISPR/Cas9 system. A CRISPR/Cas9 cocktail containing two gRNAs that targeted the *Fads1*/*2* genes and two single-stranded DNA donor templates with the loxP sequence (Supplementary Table 3) was microinjected into mouse fertilized eggs (C57BL/6JJcl strain). Nonhomologous end joining (NHEJ) resulted in the deletion of ~124 kb (*Fads*(Δ)) and homology-directed repair (HDR) generated the floxed allele. **c** Body weight of heterozygous and homozygous *Fads1/2* deficient mice. *Fads*(Δ/Δ) mice were significantly leaner than the *Fads*(Δ/+) and WT (*Fads*(+/+)) mice (*P < 0.05, *d* = 0.80 and 1.24 [large effect size (ES)], respectively, *t*-test with Bonferroni correction). Males, 18–33 weeks old. The box length and a horizontal bar show the interquartile range (IQR) and median, respectively. The length of the whiskers is defined as 1.5 times the upper and lower limits of the IQR. **d** Food intake. Daily food intake did not differ by genotype.

The *FADS1* and *FADS2* genes on a tight linkage disequilibrium (LD) block are located head-to-head in the GWAS-identified region (Supplementary Fig. 1a). These genes encode fatty acid desaturases, rate-limiting enzymes involved in the biosynthesis of ω3 (n-3) and ω6 (n-6) long-chain polyunsaturated fatty acids (PUFAs) (Fig. 1a). Linoleic acid (LA; 18:2n-6), which is abundant in the oils of grains such as corn, is converted to arachidonic acid (AA; 20:4n-6) by a two-step desaturating reaction catalyzed by FADS2 and FADS1. α-Linolenic acid (ALA; 18:3n-3), which is enriched in some seed oils such as linseed oil, is converted to eicosapentaenoic acid (EPA; 20:5n-3) by FADS2 and FADS1 and then to docosahexaenoic acid (DHA; 22:6n-3) by further unsaturation catalyzed by FADS2.

Even before this *FADS1*/*2* genomic region attracted attention as a locus of BD susceptibility shared across populations, it had seized the spotlight due to the significant changes in haplotype diversity (Supplementary Fig. 1b) since humans commenced crop agriculture [4–6]. The increased intake of grain oils has resulted in an increase in the proportion of people carrying a haplotype associated with higher FADS1/2 activity (Haplotype D) [4]. This haplotype has a protective effect against BD [1–3]. GWASs of blood lipid composition have also demonstrated a pivotal role of the *FADS1*/*2* locus in the plasma levels of ω3 and ω6 PUFAs [7]. Consistent with these results, expression quantitative trait loci (eQTL) analysis suggested that the other major haplotype (Haplotype A) conferring susceptibility to BD was associated with decreased expression of *FADS1/2* and likely with lower enzyme activity [8]. Although the odds ratio for the susceptible alleles is not high (at most 1.18) [1–3], these evolutional and functional underpinnings of the locus collectively suggest that an animal mimicking a decreased, but not completely nullified, activity of both FADS1/2 can be a valid model and contribute to a better understanding of the pathogenesis of BD. In the present study, to generate such a model, we deleted the region containing the mouse *Fads1* and *Fads2* genes in a heterozygous manner (referred to as *Fads*(Δ/+) mice).

Single knockout (KO) mice deficient in either *Fads1* or *Fads2* have been investigated prior to our study; [9–15] however, if these mice are crossed, double KO mice cannot be generated because these genes are located only approximately 100 kb apart. Additionally, since studies using single KO mice were conducted from the perspective of nutrition, they mainly analyzed homozygous KO mice fed semipurified diets [9–15]. Data on behavioral phenotypes were not reported so far.

The inverse relationship between seafood consumption and the prevalence of depression and BD highlights a possible nutritional or pharmacological effect of ω3 PUFAs, such as DHA and EPA [16]. Several previous studies reported the therapeutic efficacy of ω3 PUFAs for depressive episodes in BD [17, 18], but the results from randomized clinical trials were debatable [19]. Data on plasma PUFA levels in patients are also inconsistent; however, recent studies with the largest sample size to date have reported low levels of EPA and DHA and high levels of AA in patients with BD [20]. In addition, studies of lipids in postmortem brains are limited, with small sample sizes and inconsistent results [21–23]. Moreover, food and medication can be major confounding factors in human studies, requiring analysis using animal models that provide greater experimental control.

In this study, we established *Fads*(Δ/+) mice as a preclinical model of the GWAS-identified risk factor in BD; these mice exhibited both mania- and depression-like episodic behavioral changes. To detect these infrequent episodic behavioral changes, we monitored the wheel-running activity of *Fads*(Δ/+) mice continuously for six months. Unlike other locomotor activities, wheel running in mice is a strongly goal-directed behavior having a significant reward value [24, 25]; thus, a reduction in wheel running is associated with “markedly diminished pleasure (anhedonia)”, a core symptom of a depressive episode [26]. In addition, we provided proof of concept that long-term supplementation with DHA improved the behavioral abnormalities in the model mice.

## Methods

### Generation of *Fads1*/*2* mutant mice

All animal procedures were approved by the Wako Animal Experiment Committee of RIKEN (H27-2-233, H29-2-230, W2019-2-040, W2021-2-042). We developed *Fads*(Δ/+) and *Fads*(flox/+) mice by the CRISPR/Cas9 system, which have been deposited in RIKEN BioResource Center (RBRC11813 and RBRC11814). A detailed description of the procedure and animal husbandry is provided in Supplementary Methods.

### Determination of the lipid composition

Lipidomics analysis of brain samples was performed using liquid chromatography (LC)–tandem mass spectrometry (LC-MS/MS). All the lipidomics data were provided in Supplementary Table 1. Total fatty acid levels in plasma samples were determined by gas chromatography–mass spectrometry (GC-MS) analysis. For complete details, see Supplementary Methods.

### Behavioral testing

Recording and analyses of wheel-running activity were performed as previously described [27, 28]. Hyperactivity bouts and hypoactivity episodes were defined operationally. Tail suspension test was performed during hyperactivity bouts. IntelliCage analysis and open-field, splash, accelerating rotarod, sucrose preference tests were conducted in non-episodic periods. Detailed procedures are provided in Supplementary Methods.

### Lithium treatment and PUFA supplementation

We prepared a lithium-containing normal chow and PUFA-supplemented AIN93G diets and fed them to mice. The composition of the diets is provided in Supplementary Table 2. Since hypoactivity episode frequency was age-dependent (Supplementary Fig. 2), the effect of lithium was examined by a two-group, two-period crossover design using a cohort obtained by a single in-vitro fertilization.

### Statistics

*U*-test, *t*-test, Fisher’s exact probability test, or analysis of variance (ANOVA) was used. The Benjamin-Hochberg false discovery rate (FDR) or Bonferroni correction was applied to correct multiple comparisons. Statistical analyses were performed using Excel (Microsoft) or R (R Development Core Team). For all analyses, *P* < 0.05 was considered statistically significant. Substantive significance (effect size) was calculated using Cohen’s *d* for *t*-test, *r* for *U*-test, *η*^2^ for ANOVA, and *φ* for Fisher’s exact test. *d* > 0.01, 0.2, 0.5, and 0.8 were considered as very small, small, medium, and large effect sizes, respectively. *r* > 0.1, 0.3, 0,5; *η*^2^ > 0.01, 0.06, 0.14; and *φ* > 0.1, 0.3, 0.5 were considered as small, medium, and large effect sizes, respectively.

## Results

### Generation of *Fads*(Δ/+) and *Fads*(flox/+) mice by CRISPR/Cas9-mediated genome editing in zygotes

To model the susceptibility haplotype to BD, we generated two kinds of mutant mice by means of the CRISPR/Cas9 system: *Fads*(Δ/+) mice carrying a 124-kb genomic fragment deletion and *Fads*(flox/+) mice in which the same deletion occurs in the presence of Cre recombinase (Fig. 1b). F0 mice carrying either the *Fads*(Δ) or *Fads*(flox) allele were crossed with wild-type (WT) mice to obtain F1 mice, and we confirmed germline transmission of the genome-edited alleles. We selected strains of *Fads*(Δ/+) or *Fads*(flox/+) mice that did not harbor damaging mutations due to possible off-target effects of Cas9 or *de novo* mutagenesis through whole-exome sequencing and subsequent genotyping.

Both male and female *Fads*(Δ/+) mice were fed a normal chow diet (CRF-1 diet, Jackson Laboratory Japan), which contained fish meat components, and were fertile. *Fads*(Δ/Δ) mice were obtained by intercrossing the heterozygous mice. These *Fads*(Δ/Δ) mice weighed significantly less than WT and *Fads*(Δ/+) mice, even though they ate the similar amount of the normal chow diet (Fig. 1c, d). We did not use *Fads*(Δ/Δ) mice in this study because they completely lost FADS1/2 and therefore did not model the results of the GWAS, namely, reduced FADS1/2 activity.

We performed histological staining and found no apparent difference in gross brain structure between *Fads*(Δ/+) and WT mice (Supplementary Fig. 3). We conducted open-field, rotarod, sucrose preference, and splash tests and revealed that *Fads*(Δ/+) mice had a normal sensorimotor function and emotional response in a euthymic state (Supplementary Fig. 4). Additionally, we measured the plasma levels of seven inflammatory markers and detected no changes in *Fads*(Δ/+) mice (Supplementary Fig. 5).

**Fig. 2:**
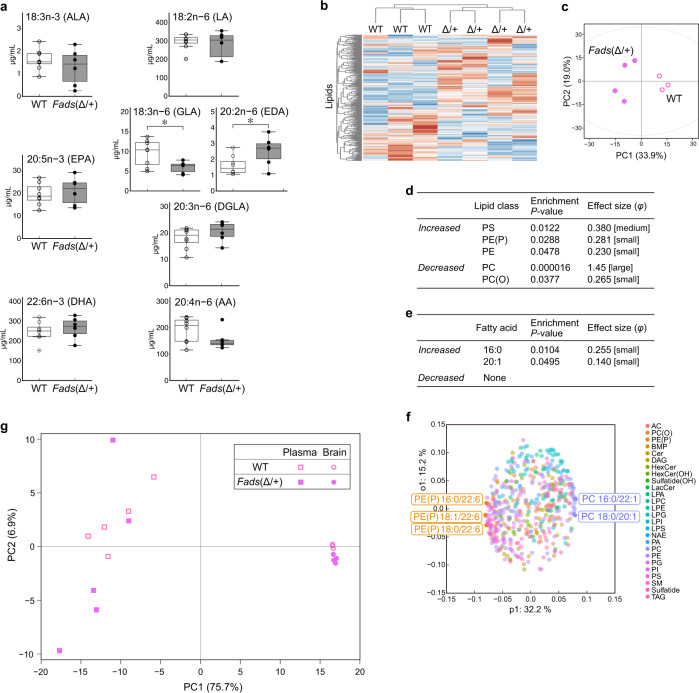
Lipid analysis of male *Fads*(Δ/+) mice fed a normal chow diet. **a** Plasma fatty acid levels in mice fed a normal chow diet. The intermediate metabolites GLA and EDA significantly differed between WT (*n* = 8) and *Fads*(Δ/+) (*n* = 6) mice (*P < 0.05, *d* = 0.658 [medium ES] and 0.272 [small ES], respectively, *t*-test with Bonferroni correction). Note that the values on the vertical axis are very different in each graph. For the boxplot description, see the legend of Fig. 1c. **b** Dendrogram of unsupervised clustering analysis of 464 brain lipids in mice fed a normal chow diet. **c** PCA plot of mice based on brain lipids. This is a part of Fig. 4b. PC1 and PC2 were calculated using all brain lipidomics data from males in this study (Fig. 4b and Supplementary Fig. 9). **d**, **e** Lipid class (**d**) and fatty acid (**e**) enrichment analysis of differentially changed brain lipids between *Fads*(Δ/+) and WT mice. Enrichment *P*-values are given by Fisher’s exact test. The abbreviations for the lipid classes are listed in Supplementary Table 4. **f** OPLS-DA loading plot of the 464 brain lipids from *Fads*(Δ/+) and WT mice. The top 1% of lipid molecules are highlighted. The comparisons between genotypes for these individual lipids are shown in Supplementary Fig. 7. **g** PCA plot of brain and plasma samples based on 282 lipids that were detected all the brain and plasma samples.

GWAS analysis on blood fatty acids revealed that the PUFA composition is affected by the genotype (or haplotype) of *FADS1/2* [7]. We measured plasma levels of total fatty acids in *Fads*(Δ/+) and WT mice by GC-MS. Intermediate metabolites in the ω6 PUFA pathway, 18:3n-6 (γ-linolenic acid, GLA) and 20:2n-6 (eicosadienoic acid, EDA), were significantly decreased and increased, respectively, but no significant change in DHA, EPA, or AA was detected (Fig. 2a). The changes in the plasma fatty acid levels in *Fads*(Δ/+) mice were similar to those observed in BD patients carrying the susceptibility allele (Saito et al., submitted).

### Lipid profiles in the brain and plasma are altered in *Fads*(Δ/+) mice fed a normal chow diet

To date, data on the brain lipid compositions are lacking in studies of single KO mice of *Fads1* and *Fads2*, especially mice fed a normal chow diet that contained DHA and EPA [9–15], as well as in studies of human patients. We thus used a targeted lipidomics approach and measured the levels of 464 lipids (26 different lipid classes, 29 types of fatty acids) by LC-MS/MS in brain samples from male *Fads*(Δ/+) and WT mice. Unsupervised hierarchical clustering and principal component analysis (PCA) based on the 464 lipids categorized *Fads*(Δ/+) and WT mice into two distinctive groups (Fig. 2b, c). To identify the lipids, lipid classes, or fatty acids that contributed to the overall difference in brain lipid composition, we looked for lipids that were altered in the mutant mice. Of the lipids measured, 70 were significantly altered with large effect sizes (*P* < 0.05 and *d* > 0.8). Lipid class enrichment analysis showed that five classes including phosphatidylcholine (PC) and phosphatidylserine (PS) significantly changed by genotype (Fig. 2d). Fatty acid enrichment analysis revealed a significant change in 16:0 and 20:1 PUFAs but not ω3 or ω6 PUFAs (Fig. 2e). We also examined the brain samples from female mice (Supplementary Fig. 6). Although female *Fads*(Δ/+) mice had a distinct brain lipid composition as compared to WT mice, the lipid classes and fatty acids that were significantly altered differed from those in males. We performed an orthogonal partial least squares discriminant analysis (OPLS-DA) to uncover individual lipid molecules contributing to the lipidomic difference between *Fads*(Δ/+) and WT mice. There were no identical molecules in the lipids identified in the comparison between males and those identified in females (Fig. 2f and Supplementary Figs. 6e, 7).

Little DHA is synthesized locally in the brain even in WT mice, and DHA in the brain mainly is derived from the blood [29]. Thus, we examined the plasma of male *Fads*(Δ/+) and WT mice in detail by LC-MS/MS and compared them with changes in brain lipids (Fig. 2g and Supplementary Fig. 8). We found that blood and brain have different lipid changes in response to reduced FADS1/2 activity, and the composition of brain lipids varied less by genotype and also less between individuals than that in the blood (Fig. 2g), probably due to robust homeostatic mechanisms in the brain [29, 30].

### Spontaneous behavioral changes, hyperactivity bouts and hypoactivity episodes in *Fads*(Δ/+) mice

BD is characterized by recurrent manic and depressive episodes [26]. To detect these infrequent episodic behavioral changes, the wheel-running activity of *Fads*(Δ/+) mice of both sexes fed a normal chow diet was recorded for six months.

**Fig. 3:**
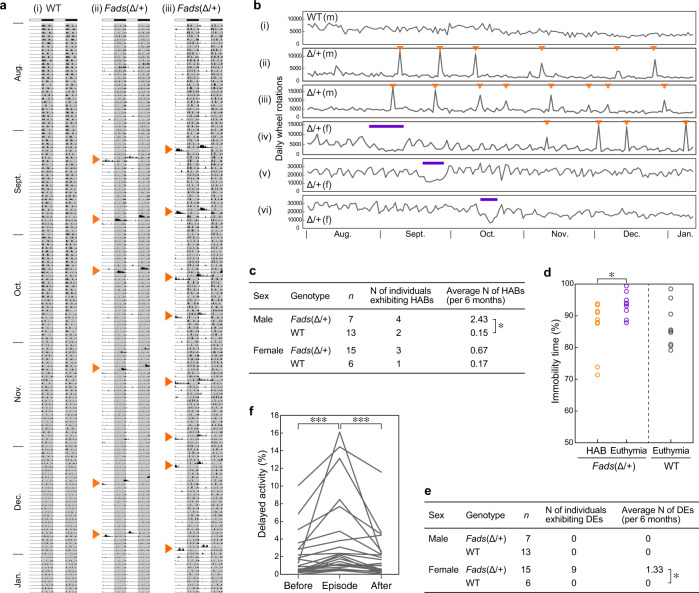
Spontaneous behavioral changes in *Fads*(Δ/+) mice fed a normal chow diet. **a** Representative double-plotted actograms of wheel-running activity in WT (i) and *Fads*(Δ/+) mice (ii, iii). Arrowheads depict hyperactivity bouts (HABs). **b** Total daily wheel-running activity for six months. Arrowheads depict HABs, and thick lines indicate depressive-like episodes. Individuals (i)–(iii) are identical to those (i)–(iii) in panel **a**, respectively. m, male; f, female. **c** Frequency of HABs. Male *Fads*(Δ/+) mice exhibited HABs significantly more often than WT mice (*P < 0.05, *r* = 0.503 [large ES], *U*-test). **d** Immobility time in the tail suspension test during HAB or the euthymic state. The immobility time of *Fads*(Δ/+) mice was significantly shorter during HABs (*P < 0.05, *d* = 1.03 [large ES], *t*-test). Data for euthymic WT mice are shown as a reference. There is a significant difference in immobility time between euthymic *Fads*(Δ/+) mice and euthymic WT mice. **e** Frequency of depression-like episodes. Female *Fads*(Δ/+) mice exhibited depression-like episodes (DEs) significantly more often than WT mice (*P < 0.05, *r* = 0.516 [large ES]). We have observed four female individuals that exhibited both DE and HAB; one of them is the individual (iv) in panel (**b**). The four animals were among 47 females that were fed a normal chow diet and examined for wheel-running behavior for more than four months. **f** Comparison of delayed activity, an indicator of abnormal circadian rhythms, during and two weeks before and after a depression-like episode. *Fads*(Δ/+) mice showed significantly higher delayed activity during episodes (***P < 0.001, *r* = 0.91 and 0.80 [large ES], paired *U*-test).

Male *Fads*(Δ/+) mice showed a marked episodic increase in wheel-running activity (Fig. 3a, b). This episodic high activity hereafter referred to as a hyperactivity bout (HAB), typically lasted approximately half a day (~6 h to 1 day). We operationally defined HABs as behavioral changes that lasted more than 6 h with sufficiently heightened activity to be considered a statistical outlier (see Supplementary Methods for details). Male *Fads*(Δ/+) mice exhibited HABs at a significantly higher frequency (~2.4 episodes in 6 months) than that of WT mice (Fig. 3c). Several female *Fads*(Δ/+) mice also exhibited HABs, but less frequently than male *Fads*(Δ/+) mice (Fig. 3c). HABs were often observed even during the light period when nocturnal mice should have been resting or sleeping (Fig. 3a).

It is difficult to verify whether the HABs in *Fads*(Δ/+) mice correspond to manic episodes. The DSM-5 criteria for a manic episode are inapplicable to mice because most of the criteria are related to subjective experiences [26]. In addition, it was impossible to predict when HABs developed, and they lasted only approximately half a day. Given the limitations of this investigation, we performed a tail suspension test during HABs, which required no prior training and only 6 min of testing time. The tail suspension test is a behavioral despair experiment that has been used in antidepressant screening (antidepressant administration shortens immobility time) and that has sometimes been used to evaluate mouse phenotype in models for depression (in general, immobility time is increased in depression models) [31]. During HABs, *Fads*(Δ/+) mice had shorter immobility times than concurrently tested littermate *Fads*(Δ/+) mice during non-episodic periods (Fig. 3d).

Female *Fads*(Δ/+) mice also exhibited several weeks of hypoactivity, and some mice showing these episodes showed HABs as well (Fig. 3b). These hypoactivity episodes were also operationally defined according to our previous study [27]. Female *Fads*(Δ/+) mice showed a significantly higher frequency of these hypoactivity episodes (~1.3 episodes in 6 months) than WT mice (Fig. 3e). As an indicator of circadian rhythm disturbances associated with the hypoactivity episodes, we used the “delayed activity index” [27], which reflects the extent to which the mice continued to run on a wheel even in the light period (i.e., morning). This index was more marked during hypoactivity episodes than during the two-week euthymic state surrounding the episodes (Fig. 3f). The frequency of hypoactivity episodes with circadian rhythm disturbances was comparable to that of depression-like episodes in mutant *Polg1* Tg mice, a mouse model of recurrent depression [27]. No such depression-like episodes were observed in male mice.

### Long-term DHA supplementation prevents depression-like episodes in *Fads*(Δ/+) mice

We investigated whether PUFA supplementation (EPA, DHA, or EPA + DHA) was effective for treating *Fads*(Δ/+) mice. We thus prepared semipurified diets supplemented with EPA and/or DHA to AIN93G that did not contain fish powder (Supplementary Table 2). Mice were raised until 25 weeks of age on a normal chow diet, and their wheel-running activity was measured at the time that they were started on the AIN93G-based diets for six months.

**Fig. 4:**
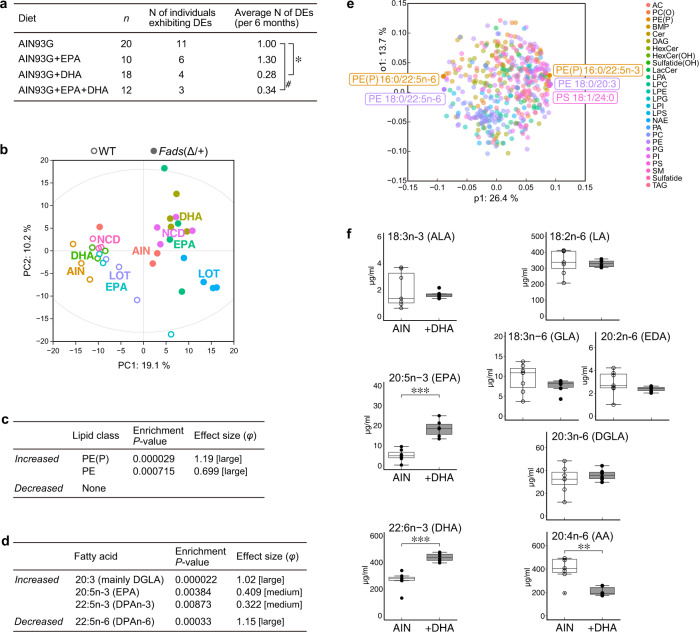
Effect of dietary DHA supplementation on the lipid compositions and behavior of *Fads*(Δ/+) mice. **a** Frequency of depression-like episodes in female *Fads*(Δ/+) mice fed the AIN93G diet (control) and AIN93G supplemented with DHA and/or EPA. DHA supplementation significantly reduced the frequency of DEs (*P < 0.05, ^#^*P* < 0.1, *r* = 0.331 and 0.315 [medium ES], *U*-test with Bonferroni correction following Kruskal-Wallis test). **b** PCA plot of *Fads*(Δ/+) and WT mice fed the five different diets based on brain lipidomics data (Supplementary Table 1a). A two-way ANOVA revealed a significant and large effect of genotype (*P* < 0.001, *η*^2^ = 0.15) but not diet on the Euclidean distance between the lipid compositions of the samples measured. **c**, **d** Lipid class (**c**) and fatty acid (**d**) enrichment analyses of differentially changed brain lipids between *Fads*(Δ/+) mice fed AIN93G and those supplemented with DHA. The abbreviations for the lipid classes are listed in Supplementary Table 4. **e** OPLS-DA loading plot of the 464 brain lipids from *Fads*(Δ/+) mice fed AIN93G and from those supplemented with DHA. The top 1% of lipid molecules are highlighted. The comparisons of these individual lipids between diet groups are shown in Supplementary Fig. 10. **f** Plasma fatty acid levels in *Fads*(Δ/+) mice fed the AIN93G diet (AIN, *n* = 7) and those of mice supplemented with DHA (*n* = 6). In *Fads*(Δ/+) mice supplemented with DHA, DHA itself and its precursor metabolite, EPA, were significantly increased (***P < 0.001, *d* = 1.884 and 1.419 [large ES], *t*-test), and the major ω6 metabolite AA was reduced (**P < 0.05, *d* = 1.437 [large ES]). For the boxplot description, see the legend of Fig. 1c.

HABs were not observed in *Fads*(Δ/+) mice of either sex fed the AIN93G control diet or the PUFA-supplemented diets. This obviously indicates that diet has a significant effect on the behavioral changes. However, depression-like episodes were observed in female *Fads*(Δ/+) mice fed AIN93G; the frequency of the hypoactivity episodes was similar to that in normal chow-fed mice (~1.0 vs. ~1.3 episodes in 6 months). The occurrence of depression-like episodes was significantly reduced in *Fads*(Δ/+) mice fed a DHA-supplemented diet compared to that in mice fed the AIN93G control diet (Fig. 4a). Supplementation with EPA + DHA that mimicked the composition of Lotriga (Takeda Pharmaceutical) also tended to reduce the frequency of these depression-like episodes. No apparent effects of the diet supplemented with EPA alone were observed.

Additionally, we examined the brain lipid composition in *Fads*(Δ/+) and WT mice fed 5 different diets (normal chow, AIN93G, AIN93G + DHA, AIN93G + EPA, and AIN93G + EPA + DHA) for 2 months. The PCA and unsupervised hierarchical clustering indicated that the effect of the *Fads1*/*2* genotype was significantly greater than the effect of the diet on the brain lipid composition (Fig. 4b and Supplementary Fig. 9). We focused on the DHA-supplemented diet, which notably prevented the depression-like episodes (Fig. 4a), and further analyzed its effect on brain lipid composition. In total, 49 lipids were altered with statistical and substantive significance in *Fads*(Δ/+) mice fed a DHA-supplemented diet compared to those of the mutant mice fed the control diet. Lipid class enrichment analysis showed that PE(P) and PE were significantly altered by diet (Fig. 4c). Fatty acid enrichment and OPLS-DA analyses revealed a significant increase in several ω3 fatty acids, such as DGLA and EPA, but in DHA (Fig. 4d, e and Supplementary Fig.10). Plasma levels of total fatty acids in these mice were also measured. DHA and EPA were increased, and AA was decreased significantly in the blood of DHA-supplemented mutant mice (Fig. 4f), indicating that lipid metabolism and homeostasis are different in the brain than the periphery.

### Lithium has a prophylactic effect on depression-like episodes in *Fads*(Δ/+) mice

**Fig. 5:**
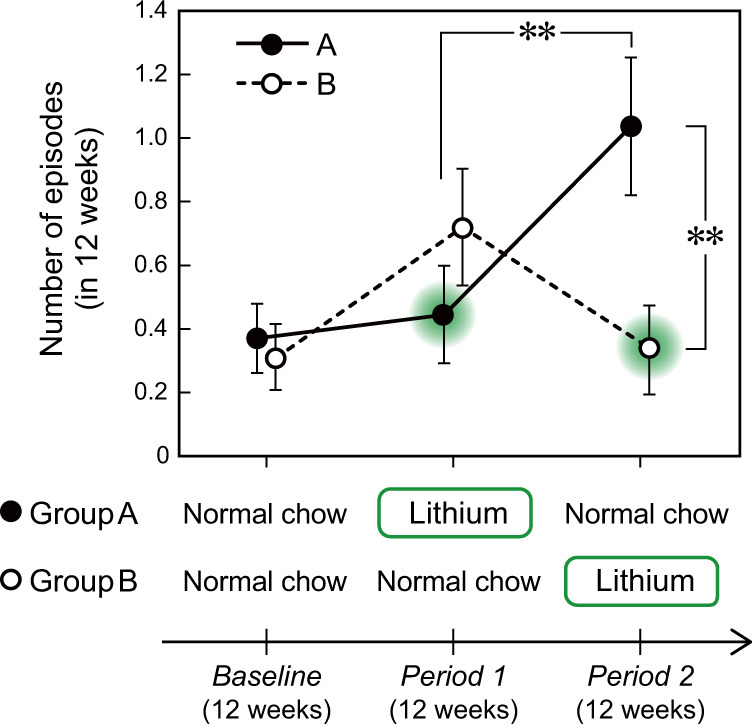
Prophylactic effect of lithium in *Fads*(Δ/+) mice. Effect of lithium treatment on the frequency of depression-like episodes. There was a significant effect of lithium treatment (*P* < 0.001, *η*^2^ = 0.061 [medium ES], two-way repeated-measures ANOVA). The episodes were significantly more frequent after treatment was terminated (Group A in Period 2) than during treatment (Group A in Period 1) (**P < 0.01, *r* = 0.583 [large ES], paired *U*-test). The episode frequency after treatment was terminated was also higher than that of another treated group (comparing Group A and Group B in Period 2) (**P < 0.01, *r* = 0.373 [medium ES], *U*-test). Data are expressed as the means ± s.e.m. Group A, *n* = 27; Group B, *n* = 25.

To evaluate the predictive validity of these mutant mice as a BD model, we tested the effects of administering lithium, a mood-stabilizing treatment for manic and depressive episodes [32]. Since it is difficult to maintain the therapeutic plasma level of lithium in male mice for months, likely due to less robustness of lithium clearance, only female mice were used in this study. To examine the effect of lithium on depression-like episodes exhibited by female *Fads*(Δ/+) mice, we administered a lithium-containing CRF-1 chow in a two-period crossover design, with each period lasting for 12 weeks (Fig. 5). After 12 weeks of baseline measurement, the mice were randomly divided into two groups (A and B). Lithium significantly decreased the frequency of depression-like episodes in these mice. In addition, we observed more frequent episodes after terminating the treatment (Fig. 5), which suggests that lithium withdrawal triggered new episodes. This finding is similar to observations in BD patients and another mouse model of mood disorders [27].

### Brain-specific deletion of *Fads1*/*2* had no significant impact on behavioral phenotypes

In the brain, *Fads1* is expressed in both neurons and glia, while *Fads2* is expressed mainly in glial cells and subsets of neurons according to the Single Cell Portal [33]. Since these enzymes are also widely expressed in peripheral, non-neural tissues, we next examined whether the HABs and depression-like episodes in *Fads*(Δ/+) mice were caused by abnormal PUFA metabolism in the brain or by peripheral metabolic disorders. We crossed *Fads*(flox/+) mice with Nestin-Cre (*NC*) mice (a brain-specific Cre driver) and obtained brain-specific conditional knockout (cKO) mice of the genotype *Fads*(flox/+);*NC*/+, in which *Fads1/2* genes were heterozygously deleted in ~80% of cells in the brain (Supplementary Fig. 11). These mice and control *Fads*(+/+);*NC*/+ mice were fed a normal chow diet, and their wheel-running activity was measured for six months. Neither male nor female cKO mice exhibited HABs or depressive-like episodic behavioral changes, in contrast to *Fads*(Δ/+) mice (Supplementary Fig. 12). No sleep-wake rhythm abnormalities were detected either (Supplementary Fig. 13). To detect non-episodic behavioral phenotypes, we performed a battery of behavioral tests assessing place learning ability, impulsivity, attention control, *etc*. using the IntelliCage [34–36]. We compared *Fads* cKO mice of both sexes fed a normal chow diet (under group-feeding conditions) compared with controls and observed that none of the tested behaviors displayed any differences (Supplementary Fig. 14). Moreover, there were no significant changes in the plasma fatty acid levels of *Fads* cKO mice compared with those of the controls (Supplementary Fig. 15). These results suggest that the behavioral abnormalities in *Fads*(Δ/+) mice (i.e., the HABs and depression-like episodes) were due not to reduced FADS1/2 activity in the neurons or astrocytes but to reduced FADS1/2 activity in the periphery or microglia and endothelial cells in the brain where *NC* did not work.

## Discussion

In this study, we focused on the *FADS1/2* gene region, which was identified in large-scale GWASs of BD in multiple populations [1–3]. We generated heterozygous KO mice (*Fads*(Δ/+)) to clarify the functional relevancy of the genes and the susceptibility alleles to BD. Using behavioral and lipidomics approaches, we confirmed that they have construct, face, and predictive validity [37] as an animal model of BD. Its high construct validity is conferred by heterozygous deletion of the *Fads1*/*2* gene, which mimics the reduced FADS1/2 enzyme activity observed in the BD risk haplotype. Previously proposed mouse models of mania [38, 39], such as *Clock* mutant mice and methamphetamine-treated mice, had a certain level of construct validity, but most of them lacked episodic phenotypes. In contrast, *Fads*(Δ/+) mice exhibited episodic behavioral changes, depression-like episodes and HABs (Fig. 3a, b). However, it is fundamentally difficult to evaluate HABs in mice using the DSM-5 diagnostic criteria for manic episodes because the primary criterion is an abnormally and persistently elevated mood, which is a subjective perception or experience. Other diagnostic criteria, such as inflated self-esteem and flight of ideas, are also difficult to evaluate in mouse models. However, the HABs of *Fads*(Δ/+) mice are considered to meet the following DSM-5 diagnostic criteria for manic episodes: a decreased need for sleep and an increase in goal-directed activity. This is because the mutant mice exhibited sustained wheel-running activity even in the light phase during days of HABs (Fig. 3a); wheel running is a goal-directed behavior in rodents and is closely linked to the reward system [24, 25, 27]. In addition, these mice exhibited decreases in immobility time in the tail suspension test during HABs (Fig. 3d), which supports the idea that the HAB is a mania-like episode. *Fads*(Δ/+) mice also spontaneously showed hypoactive episodes that lasted for two weeks or more (Fig. 3b). This behavioral phenotype was accompanied by abnormal circadian rhythms (Fig. 3f) and is very similar to the hypoactive episodes exhibited by mutant *Polg1* Tg mice, which satisfied the DSM-5 criteria for depressive episodes [27, 28]. To the best of our knowledge, these findings indicate that the *Fads*(Δ/+) mouse model has the most clinically relevant face validity for BD to date.

Regarding the predictive validity of this model, we demonstrated that DHA supplementation (and also DHA + EPA supplementation) was effective in reducing the frequency of depression-like episodes (Fig. 4a). Comprehensive lipid analysis showed that DHA supplement slightly altered the brain lipid composition of *Fads*(Δ/+) mice, but did not alter the level of DHA, nor did it alter the lipid composition to resemble that of WT mice (Fig. 4b and Supplementary Fig. 16). This result may reflect strong homeostasis to maintain constant PUFA levels, especially DHA in the brain. Nevertheless, DHA supplement exerted the prophylactic effect (Fig. 4a), possibly because it facilitated the homeostatic responses to lower FADS1/2 activities in *Fads*(Δ/+) mice. Although seemingly contrary to the results of the meta-analysis of clinical studies that EPA supplement, rather than DHA, is more effective in treating BD [17, 18, 40], clinical studies include the problem of not being able to control diet and DHA supplement could be particularly effective in BD patient with *FADS1/2* risk allele. Lastly, we emphasize that lithium treatment also had a prophylactic effect in these mice (Fig. 5), which further supports the model’s predictive validity.

One of the limitations of the model is sex differences in the behavioral phenotypes. Male mice did not experience depression-like episodes, and female mice exhibited less frequent HABs than males (Fig. 3c, e). This appears to be inconsistent with the lack of substantial sex difference in the prevalence of BD [41]. A recent paper, however, reports that depressive episodes are more frequent in female patients with BD than in males, which would be consistent with the fact that BD-II is more prevalent in females [42]. Thus, we would need to investigate the sex ratio of BD patients who have the susceptibility allele, as well as their detailed symptoms, in addition to studying the mechanism underlying the sex differences in *Fads*(Δ/+) mice. Another limitation is that we have not been able to evaluate the efficacy of lithium on male *Fads*(Δ/+) mice or the effects of other therapeutic drugs because of the lack of a chronic administration regimen over a period of several months. Because of the infrequency of episodic behavioral changes exhibited by this model (Figs. 3–5), there is an urgent need to establish methods for the long-term administration of various psychotropic drugs to mice.

Curiously and importantly, brain-specific cKO mice lacked apparent behavioral phenotypes (Supplementary Figs. 12–14). It suggests that the reduced activity of FADS1/2 enzymes in peripheral tissues primarily leads the BD-like episodic behavioral change in *Fads*(Δ/+) mice. The contribution of microglia and endothelial cells in the brain, in which *Nestin*-Cre does not work in cKO mice, cannot be ruled out, but this is unlikely because the expression of *Fads1/2* is very low. In the systemic KO mice, plasma inflammatory markers were unaltered (Supplementary Fig. 5), but blood lipid composition was altered to a greater extent than in the brain (Fig. 2g). Blood levels of fatty acids were completely unchanged in cKO mice (Supplementary Fig. 15). Even in animal studies, it will be necessary to study BD not only as a disease of the brain but also as a disease of the whole body. *Fads*(Δ/+) mice, which satisfy all three validities, will particularly help us to understand the pathogenesis of BD and develop therapeutic interventions in patients carrying the *FADS1*/*2* susceptibility allele (approximately half of all patients with BD).

### Supplementary information

Supplementary Methods

Supplementary Figures

Supplementary Table1

Supplementary Table2

Supplementary Table3

Supplementary Table4
