# Supplementary material for: GWAS-identified bipolar disorder risk allele in the FADS1/2 gene region links mood episodes and unsaturated fatty acid metabolism in mutant mice
Source: Mol Psychiatry. 2023 Feb 21;28(7):2848–56. doi: 10.1038/s41380-023-01988-2 (PMC10615742; doi:10.1038/s41380-023-01988-2)
Supplement: Supplementary file 1 — Supplementary Methods [file 41380_2023_1988_MOESM1_ESM.docx]

**Supplementary Methods**

## Animals and husbandry

All animal procedures were approved by the Wako Animal Experiment Committee of RIKEN (H27-2-233, H29-2-230, W2019-2-040, W2021-2-042). Mice were kept in humidity- and temperature-controlled rooms (23 ± 1 °C, 50 ± 5% humidity) with food and water provided *ad libitum*. Unless otherwise noted, mice were fed a normal chow diet (CRF-1, Jackson Laboratory Japan). Mice were almost always group-housed (3–5 per cage) at weaning in polycarbonate cages (26 × 16 cm) and maintained at a 12 h:12 h light/dark cycle, with 50–200 lux illumination at the level of the cage.

*Fads*(Δ/+) mice were always used as heterozygotes, which were produced by mating wild-type (WT) C57BL/6J females with mutant males, and the controls were WT littermates or WT mice obtained at the same *in*-*vitro* fertilization (IVF). *Fads*(flox/+);*NC*/+ mice were produced by *in-vitro* fertilization to cross *Fads*(flox/+) line with Nestin-Cre (*NC*) driver line [B6.CgTg(Nes-cre)1kln/J] (obtained from the Jackson Laboratory). The controls were *Fads*(+/+);*NC*/+ littermates or *Fads*(+/+);*NC*/+ mice obtained at the same IVF.

## Generation of *Fads*(Δ/+) and *Fads*(flox/+) mice by the CRISPR/Cas9 system

To efficiently cleave the second half (or the end) of the *Fads1* and *Fads2* genes, which are located in head-to-head, by *S.p.* Cas9, we designed three crRNA sequences for each locus using several online tools and selected one for each based on the results of the Surveyor assay. Briefly, we microinjected each of the candidate crRNA sequences, tracrRNA, and Cas9 protein (all from Integrated DNA Technologies, Coralville, IA, USA) into fertilized mouse eggs and analyzed the indels introduced into the embryo genomes with Guide-it Mutation Detection Kit (Takara Bio). The target sequences of the selected crRNAs were as follows: crRNA_Fads1, 5´-TAGGATGCCACTTCACG|CAGggg-3´, which induces cleavage at the base pair [Chr19:10,165,598 (mm39)] indicated by "|" in intron 5; and crRNA_Fads2, 5´-TAGGGCTCACCACTTAC|CACagg-3´ (Chr19:10,041,815) in intron 11.

crRNA_Fads1, crRNA_Fads2, and tracrRNA were annealed (molar ratio 1:1:2) by heating at 95 °C for 5 min and cooling to room temperature in Duplex buffer (Integrated DNA Technologies). These RNAs (at final concentrations of 5, 5, and 10 µM) were mixed with ssDNA_Fads1 (final 10 ng/µL), ssDNA_Fads2 (final 10 ng/µL), and Cas9 protein (final 100 ng/µL), centrifuged at 21,200 × *g* for 20 min at 4 °C, and incubated at room temperature for 15 min. The sequences of ssDNA_Fads1 and ssDNA_Fads2, which were used as homology-directed repair (HDR) templates containing the loxP sequence, are shown in Supplementary Table 3. The mixture was microinjected into the pronucleus of fertilized eggs (C57BL/6JJcl strain; CLEA Japan, Tokyo, Japan). Among the 999 eggs injected, 477 embryos developed into blastocysts *in vitro* and were transferred to recipient dams, resulting in 70 F0 mice. Genotyping PCRs (for details, see the next section) and sequencing of the PCR products revealed that at least 6 F0 mice carried the loxP sequence in both *Fads1* and *Fads2* (*Fads*(flox)) resulting from HDR, and at least 17 F0 mice carried a large deletion (*Fads*(Δ), ~124 kb) resulting from nonhomologous end joining (NHEJ) between the cleavage sites of *Fads1* and *Fads2*.

We crossed the F0 mice with C57BL/6J mice and thereby obtained 5 *Fads*(flox/+) strains and 2 *Fads*(Δ/+) strains of F1 mice. We performed whole-exome sequencing essentially as previously described [1] to determine whether the F1 mice carried damaging mutations due to off-target activity of Cas9 or *de novo* mutagenesis. Compared to the parental strain (C57BL/6JJcl), one F1 *Fads*(flox/+) mouse carried one heterozygous protein-altering mutation in the *C8a* gene (Chr 4), which was predicted to be neutral by the PROVEAN and SIFT programs. One F1 *Fads*(Δ/+) mouse carried three heterozygous protein-altering mutations in the *D930015E06Rik* (Chr 3), *Matn4* (Chr 2), and *Neto1* (Chr 18) genes. The first mutation was predicted to be neutral, but the others (V143F in *Matn4* and R369P in *Neto1*) were predicted to be damaging mutations. We confirmed that these deleterious mutations were eliminated in the third generation of the *Fads*(Δ/+) line and used only the fourth or later generations for behavioral and biochemical analyses.

## Genotyping

Genomic DNA was isolated from tail biopsies. Genotyping for the *Fads*(Δ) allele was performed by PCR using three primers: Fads1_Rv, 5´-ATGCTGATCACGCTGAAAGAC-3´; Fads2_Fw, 5´-AGTGGGGATTCCTGTCTACCC-3´; and Fads2_Rv, 5´-CAGGAACCAGATGGAAGGAAC-3´. *Fads1*(flox) and *Fads2*(flox) alleles were tested by PCR using the following primers: Fads1_Fw, 5´-AACCTGGGTTACATAGCAAGACC-3´ and Fads1_Rv (detailed above); and Fads2_Fw and Fads2_Rv (detailed above). The Nestin-Cre transgene was genotyped by PCR using three primers: NesCre-WTF, 5´-CAGCCAGCATAATTTGCTAAAG-3´, NesCre-WTR, 5´-TGGATTATAAAGCTCCAATCAGG-3´, and NesCre-TgR, 5´-TCCAGCTTGGTTCCCAATAG-3´. After crossing *Fads*(flox) and NesCre mice, we genotyped the *Fads* locus by PCR using three primers: Fads1_Rv, Fads2_Rv (detailed above), and Fads2_Fw2, 5´-CCCTGATCGACATTGTGAGG-3´.

**Digital PCR**

A hemisphere of the mouse brain was homogenized in phosphate-buffered saline containing 3 mM ethylenediaminetetraacetic acid, and genome DNA was extracted from the homogenate using Monarch Genomic DNA Purification Kit (Cat# T3010S, New England Biolabs). We performed digital PCR using the QuantStudio 3D system (Thermo Fisher Scientific) according to the manufacturer's procedure. We used TaqMan Copy Number Reference Assay, mouse, Tfrc (Cat# 4458366, Thermo Fisher Scientific) to count the Tfrc gene on the chromosome 16 (2 copies per the diploid genome) and TaqMan Copy Number Assays (Assay ID: Mm00629228_cn and Mm00629231_cn, respectively) to count the Fads2 exon 1 and Fads3 exon 1 regions, respectively. The results showed that the amount of *Fads2* gene was 52.7% as expected in ubiquitous heterozygous KO mice and 60.1% in the *Fads*(flox/+);*NC*/+ mice. This indicates that in the brains of *Fads*(flox/+);*NC*/+ mice, almost all neurons and glial cells were heterozygously deficient in *Fads1/2* (Supplementary Fig. 11).

**Determination of the fatty acid composition in plasma**

Mice were anesthetized with isoflurane, and blood was collected from the heart by needle aspiration in a 1-mL syringe filled with 5 μL of 0.5 M EDTA-Na_2_ (pH 8.0). The whole blood was centrifuged at 1,000 × *g* for 5 min, and the supernatant (plasma) was collected and stored at -80 or -20 °C until analysis. Fatty acid levels were determined by gas chromatography–mass spectrometry (GC-­MS) analysis. We added 25 μL of 0.2 mg/mL stable isotope-labeled heptadecanoic acid (METHYL-D3)/methanol to 50 μL of plasma. Methyl esterification and purification of fatty acids were conducted with a fatty acid methylation kit (Nacalai Tesque, Kyoto, Japan; #06482-04) and a methylated fatty acid purification kit (Nacalai Tesque, #06483-94). The methyl-derivatized fatty acid sample was concentrated to approximately 0.3 mL through exposure to flowing nitrogen gas and assayed using GCMS-TQ8040 (Shimadzu, Kyoto, Japan) equipped with an SP-2560 Capillary GC Column (Sigma-Aldrich Supelco, #24056) using helium as carrier gas. The concentration of each fatty acid was calculated by the peak area ratio of the internal standard to the analyte.

**Determination of the lipid composition of the brain and plasma**

Half of the mouse brain was ground to homogeneity in 5 mL of methanol/water (2:0.7 [v/v]) solution using a glass homogenizer and scaled up to 10 mL of methanol/water (2:0.7 [v/v]) solution. An aliquot (50 µL) of the homogenized solution was used for analysis. For lipid analysis of plasma, 10 µL of EDTA-treated plasma was used. Lipids were extracted using the Bligh and Dyer method with internal standards. The organic (lower) phase was transferred to a clean vial and dried under a nitrogen stream. The lipids were resolubilized in methanol and stored at -80 °C. A portion of the extracted lipids was injected into an ultrahigh-performance liquid chromatography (LC)-electrospray ionization (ESI)–tandem mass spectrometry system (LC-MS/MS). The quantification of FFA was carried out as described previously [2]. For the quantification of LPA, LPG, LPI, LPS, PA, and PS, LC separation was performed on a 50 × 4.6 mm 5 µm Gemini C18 column (Phenomenex, Torrance, CA, USA) coupled to a guard column (Gemini; C18; 4 × 3.0 mm; Phenomenex SecurityGuard cartridge). Mobile phase A was H_2_O/methanol = 95/5 (v/v%), mobile phase B was isopropanol/methanol = 63/37 (v/v%), mobile phase C was H_2_O/methanol/28% NH_4_OH = 93/5/2 (v/v/v%). The LC method consisted of 0.1 mL/min of A/C = 95/5 (v/v%) for 5 min, 0.4 mL/min linear gradient to B/C = 95/5 (v/v%) over 15 min, 0.5 mL/min B/C = 95/5 (v/v%) for 8 min, and equilibration with 0.4 mL/min A/C = 95/5 (v/v%) for 5 min (33 min total run time). The column temperature was 25 °C. For the quantification of BMP, an isocratic LC separation was performed with methanol containing 10 mM ammonium formate on a COSMOCORE 2.6C18 column (2.1 × 100 mm; Nacalai Tesque) coupled to an ACQUITY UPLC BEH C18 VanGuard Pre-column (1.7 µm, 2.1 × 5 mm; Waters, Milford, MA, USA). The flow rate was 0.3 mL/min, and the column temperature was 55 °C. For the quantification of other lipid classes, LC separation was performed on an ACQUITY UPLC BEH C18 column (1.7  µm, 2.1 × 100  mm; Waters) coupled to an ACQUITY UPLC BEH C18 VanGuard Pre-column (1.7  µm, 2.1 × 5  mm; Waters). Mobile phase A was acetonitrile/water = 60/40 (v/v%) containing 10 mM ammonium formate and 0.1% (v/v) formic acid, and mobile phase B was isopropanol/acetonitrile = 90/10 (v/v%) containing 10 mM ammonium formate and 0.1% (v/v) formic acid. The LC gradient consisted of 20% B for 2 min, a linear gradient to 60% B over 4 min, a linear gradient to 100% B over 16 min, and equilibration with 20% B for 5 min (27 min total run time). The flow rate was 0.3 mL/min, and the column temperature was 55 °C. Multiple reaction monitoring (MRM) was performed using a Xevo TQ-S micro triple quadrupole mass spectrometry system (Waters) equipped with an ESI source. The ESI capillary voltage was set at 1.0 kV, and the sampling cone was set at 30 V. The source temperature was 150 °C, the desolvation temperature was 500 °C, and the desolvation gas flow was 1,000 L/h. The cone gas flow was 50 L/h.

We detected 464 lipids from a male mouse brain (Supplementary Table 1a) and used data- and knowledge-driven approaches to compare the lipidomics data from, for example, *Fads*(Δ/+) and WT fed normal chow. For the data-driven approach, we performed an unsupervised hierarchical clustering and a principal component analysis to compare the lipidomics data overall and an orthogonal partial least squares discriminant analysis to compare individual lipids, by using lipidr package version 2.6.0 (https://github.com/ahmohamed/lipidr) [3] with the default parameters. The Euclidean distance was calculated using the R dist function. The knowledge used in the knowledge-driven approach is that each lipid belongs to one of 26 lipid classes (Supplementary Table 4) and that each lipid consists of one to three fatty acids (29 fatty acids were detected in the LC-MS/MS analysis). First, from the 464 lipids, we extracted the lipids whose amounts significantly altered with large effect sizes (*P* < 0.05 and *d* > 0.8) in comparison between *Fads*(Δ/+) and WT. We then used Fisher's exact test to determine whether the 70 lipids were enriched in a particular lipid class more often than expected by chance or bound to a particular fatty acid more frequently than by chance. The lipidomics data for male mouse brains, female mouse brains, and male mouse plasma samples are presented in Supplementary Table 1a­–c, respectively. Commonly detected lipids both plasma and brain in male mice are listed in Supplementary Table 1d.

**Long-term recording of wheel-running activity**

Recording and analyses of wheel-running activity were performed as previously described [4,5]. Mice were individually housed in cages (24 cm wide × 11 cm deep × 14 cm high) equipped with a running wheel (5 cm wide × 14 cm in diameter). The LD cycle was 12 h:12 h (lights on at 8:00 local time, which is defined as Zeitgeber time [ZT] 0; and lights off at 20:00, or ZT 12) and controlled by a PC computer system (O’Hara & Co., Tokyo, Japan). The calendar day of the wheel-running analysis started at ZT 0 (8:00 local time) and ended at ZT 24 (8:00 of the next day). Illumination was provided by a white LED at an intensity of 20–50 lux at the level of the mouse’s eyes in the cage. Food and water were available *ad libitum* unless otherwise specified. Wheel-running activity was recorded by an online PC computer system (O’Hara & Co.).

**Definition of hyperactivity bouts and hypoactivity episodes**

To mathematically define hyperactivity bouts (HABs), we applied Grubbs' test statistic ($G$), which is used to detect outliers in a univariate data set. This definition was applied because determination of whether the wheel-running activity on a given day is significantly higher than that on other days depends on the results of comparisons with the activity on other days.

For each hour of a given day, the Grubbs statistic ($G_{ZT}$) was calculated based on the wheel-running activity during the same hour (ZT) over the 8 days (equivalent to two estrous cycles) before and the 8 days after (in total, 17 days, including the given day) as follows:

$$G_{ZT}=\frac{X_{ZT}-\bar{X_{ZT}}}{s_{ZT}}, ZT=0,...,23$$

where $X_{ZT}$, $\bar{X_{ZT}}$, and $s_{ZT}$ denote the wheel-running activity in the specified hour (ZT) of the given day, the 17-day average of wheel-running activity in the ZT, and the corrected sample standard deviation, respectively. If $G_{ZT}>2.475$, which corresponds to $\alpha<0.05$ in the Smirnov‐Grubbs test (one-sided), the wheel-running activity in the ZT of the given day was considered an outlier. In addition, if the wheel-running activity in the specified hour (ZT) was greater than the average activity per hour over the 17 days (17 × 24 = 2,448 h total), the ZT of the given day was considered an outlier hour. If the daily total of the sum of the $G_{ZT}$ of outlier hours ($H$) exceeded 14.85 (= 2.475 × 6), then the activity of that day was defined as a HAB.

$$H=\sum_{\begin{aligned} ZT=0 \\ ZT\neq outlier hour \end{aligned}}^{23} G_{ZT}$$

This threshold (14.85) estimates that abnormally higher activity should continue for 6 h. However, if 5 h of extreme outliers ($G_{ZT}\approx3$) were recorded in a day, this threshold could also be exceeded. In the case of heightened activity over two days, if the sum of $H_{Day1}$ and $H_{Day2}$ exceeds the threshold, the definition of a HAB was met.

We determined hypoactivity episodes using the same criteria as previously described, which was based on the Relative Strength Index [5].

**Delayed activity index** The delayed activity index is defined as the proportion of activity during the first 3 h of the light period (ZT 0–3, or 8:00–11:00 local time) relative to the total activity during the previous day (ZT 0–24, or 8:00–8:00 local time) [4].

**Open-field test**

Open-field test was performed using 16 male (*Fads*(Δ/+), *n* = 8; WT, *n* = 8) and 14 female mice (*Fads*(Δ/+), *n* = 6; WT, *n* = 8). The mice were placed in an experimental room and allowed to habituate for 30 min. Each mouse was placed gently one edge of the open-field arena (60 cm × 60 cm) (O’Hara & Co.). The color of the floor and walls (30 cm high) was gray. A white LED-based planar lighting was placed on the ceiling (75 cm from the arena floor) and was set to 70 lux at the center of the open-field arena. The spontaneous behaviors of mice in the arena were videotaped for 1 hour using a CCD camera mounted on the ceiling.

**Splash test**

One week after the open-field test, the same mice were subjected to a splash test. Mice were placed in the outside of home cage and sprayed a 10% sucrose solution on the dorsal coat. After confirming that mice have been sprayed enough to see a thin layer of sucrose solution mist covering the entire dorsal coat, mice were returned to their home cages and videotaped their behavior for 5 min. Duration of grooming their face and back were measured manually with a stopwatch while watching the recorded videos.

**Accelerating rotarod test**

One week after the splash test, the same mice were subjected to an accelerating rotarod test. The mice were placed in an experimental room and allowed to habituate for 30 min. Mice were placed on a rotarod apparatus (MK-670; Muromachi Kikai Co., Tokyo, Japan) accelerating from 4 to 40 rpm in 5 min. Each trial ended when the mouse fell off the rod, and latency was recorded. Mice were tested four trials a day (1 h interval) for 3 consecutive days.

**Sucrose preference test**

Sucrose preference test was conducted using 24 males (*Fads*(Δ/+), *n* = 16; WT, *n* = 8) and 14 females mice (*Fads*(Δ/+), *n* = 8; WT, *n* = 6) while observing their behavior in a wheel-running apparatus. Mice were given a free choice between two bottles, one with 0.75% sucrose solution and another with water. The mice were continuously housed in cages with a wheel. To control for side preference in drinking behavior, the position of the bottles was switched every 24 h. The consumption of water and sucrose solution was measured by weighing the bottles every day at the same time of the day for 5 consecutive days. The preference for sucrose was calculated as a percentage of consumed sucrose solution of the total amount of liquid drunk.

**Tail suspension test during hyperactivity bouts**

We performed this experiment twice, each time with mice obtained by a single IVF. In total, 43 male *Fads*(Δ/+) mice and 14 male WT mice were used. We recorded their wheel-running activity for 30 weeks as described above and conducted the tail suspension test when an animal exhibited a HAB. On the same day, we also tested a *Fads*(Δ/+) littermate in a non-episode period and a WT littermate (in a non-episode period) as controls. If these littermates were not available, we tested other mice of similar ages.

The tail suspension test was carried out at ZT 4–6 (12:00–14:00 local time). Each mouse was suspended by its tail via a small aluminum plate that was attached to the tail ~1 cm from the base with adhesive medical tape; the plate was hooked to an attachment located inside an opaque box (48 × 22 × 33 cm) (O’Hara & Co.). The distance between the tip of the nose of each mouse and the floor was ~15 cm. The behaviors of the mice were videotaped for 6 min using a CCD camera mounted in the box. The recorded video data were analyzed using an online/offline PC computer system (O’Hara & Co.). The analyses were performed in an essentially blinded manner as to mouse state (HAB or euthymia).

**Long-term dietary supplementation with PUFAs**

We prepared three AIN93G-based diets (supplemented with EPA, DHA, and a mixture of EPA and DHA). The composition of the diets is provided in Supplementary Table 2. The dose of EPA, DHA, or the mixture based on dietary intake was approximately six times higher than that used for patients (Lotriga 2 g/day), but approximately half of the human equivalent dose based on the body surface area of mice. EPA and DHA were purchased from Nu-Chek Prep, Inc. (Elysian, MN, USA), and the linseed and soybean oils were obtained from Oriental Yeast Co. (Tokyo, Japan). During the long-term dietary supplementation, animals were fed once a week in amounts that were almost entirely consumed by the end of the week.

**Long-term lithium treatment**

To administer long-term (> 10 weeks) lithium treatment for mice at the therapeutic plasma level shown in humans, we fed female mice a LiCl-containing diet (1.7 g/kg) and a salt solution (450 mM NaCl) as well as water, as previously described [5]. It was difficult to maintain the therapeutic plasma level of Li^+^ in male mice. Male mice in particular seemed to have a higher clearance capacity, and using the same lithium concentration as females, it took more than a month to raise plasma lithium concentrations sufficiently. On the other hand, feeding higher lithium-containing diets quickly led to renal malfunction and many individuals became lethal due to lithium toxicity. In addition, even when fed the same amount of lithium as the females, about 15% of the individuals became lethal or unable to run on a wheel. The female mice were obtained through a single IVF by a two-group, two-period crossover design. At 20 weeks of age, the wheel-running measurement was initiated. After the initial 12-week baseline measurement, the mice were randomly divided into two groups (A and B). Mice in Group A (*n* = 27) were fed a lithium-containing diet for the first 12 weeks (Period 1) followed by a normal diet (no supplemental lithium) for 12 weeks (Period 2). Mice in Group B (*n* = 25; we originally assigned 27 animals, but two died during the 36-week experiment) were fed a normal diet in Period 1 and a lithium-containing diet in Period 2. Both treated and untreated mice were provided with two bottles: one with 450 mM NaCl solution and one with water. The mice were checked regularly to make sure they had plenty of water and that the cages were dry.

**IntelliCage analysis**

The IntelliCage apparatus and software (NewBehavior AG, Zurich, Switzerland) have been previously described [1,6–8]. The procedure was performed in a similar method as the previous reports [1]. The tasks were performed with both male and female mice obtained by one IVF. A radiofrequency identification transponder (Faread Technology Co., Ltd. Zhejiang, China) was subcutaneously implanted into the dorsocervical region of the mice under isoflurane inhalation anesthesia. Subsequently, the mice were allowed to recover for at least 1 week. During all adaptation phases and tasks in the IntelliCage system, the mice were fed normal chow and maintained on synthetic bedding (ALPHA-dri, Shepherd Specialty Papers, Watertown, TN, USA) that was changed every 2–3 weeks depending on the task schedule. Lights were on between 8:00 and 20:00. The schedule of tasks was performed in the order shown in Supplementary Fig. 14a. In the delay discounting test, the preference for saccharin water in the control mice was much stronger than previously observed [1,9]. We excluded these data because it was difficult to assess the delay discounting functions.

**Supplementary References**

1. Nakamura T, Nakajima K, Kobayashi Y, Itohara S, Kasahara T, Tsuboi T, et al. Functional and behavioral effects of de novo mutations in calcium-related genes in patients with bipolar disorder. Hum Mol Genet. 2021;30: 1851–1862.

2. Lee-Okada H-C, Hama K, Yokoyama K, Yokomizo T. Development of a liquid chromatography-electrospray ionization tandem mass spectrometric method for the simultaneous analysis of free fatty acids. J Biochem. 2021;170: 389–397.

3. Mohamed A, Molendijk J, Hill MM. lipidr: A Software Tool for Data Mining and Analysis of Lipidomics Datasets. J Proteome Res. 2020;19: 2890–2897.

4. Kasahara T, Kubota M, Miyauchi T, Noda Y, Mouri A, Nabeshima T, et al. Mice with neuron-specific accumulation of mitochondrial DNA mutations show mood disorder-like phenotypes. Mol Psychiatry. 2006;11: 577–593.

5. Kasahara T, Takata A, Kato TM, Kubota-Sakashita M, Sawada T, Kakita A, et al. Depression-like episodes in mice harboring mtDNA deletions in paraventricular thalamus. Mol Psychiatry. 2016;21: 39–48.

6. Krackow S, Vannoni E, Codita A, Mohammed AH, Cirulli F, Branchi I, et al. Consistent behavioral phenotype differences between inbred mouse strains in the IntelliCage. Genes Brain Behav. 2010;9: 722–731.

7. Endo T, Maekawa F, Võikar V, Haijima A, Uemura Y, Zhang Y, et al. Automated test of behavioral flexibility in mice using a behavioral sequencing task in IntelliCage. Behav Brain Res. 2011;221: 172–181.

8. Masuda A, Kobayashi Y, Itohara S. Automated, long-term behavioral assay for cognitive functions in multiple genetic models of Alzheimer’s disease, using IntelliCage. J Vis Exp. 2018;138: 58009.

9. Kato TM, Kubota-Sakashita M, Fujimori-Tonou N, Saitow F, Fuke S, Masuda A, et al. Ant1 mutant mice bridge the mitochondrial and serotonergic dysfunctions in bipolar disorder. Mol Psychiatry. 2018;23: 2039–2049.
