# Supplementary material for: GWAS-identified bipolar disorder risk allele in the FADS1/2 gene region links mood episodes and unsaturated fatty acid metabolism in mutant mice
Source: Mol Psychiatry. 2023 Feb 21;28(7):2848–56. doi: 10.1038/s41380-023-01988-2 (PMC10615742; doi:10.1038/s41380-023-01988-2)
Supplement: Supplementary file 2 — Supplementary Figures [file 41380_2023_1988_MOESM2_ESM.pdf]

Supplementary Figure 1

a

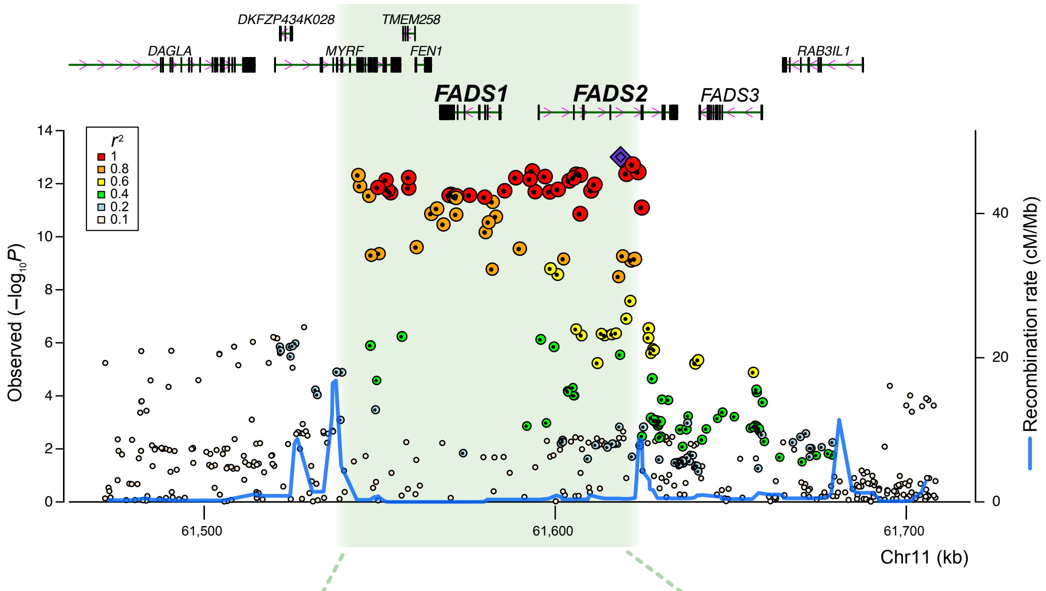

b

SNP ID	rs174544	rs174545	rs174546	rs174547	rs174548	rs174549	rs174550	rs174551	rs174553	rs174554	rs174555	rs174556	rs174560	rs174561
Modern human Haplotype D	C	C	C	T	C	G	T	T	A	A	T	C	T	T
Modern human Haplotype A	A	G	T	C	G	A	C	C	G	G	C	T	C	C
Neanderthal	C	G	C	C	G	G	C	T	G	A	T	C	C	C
Denisovan	C	G	T	C	G	A	C	–	G	G	C	T	C	C
Chimpanzee	A	G	T	C	G	A	–	C	G	G	C	T	C	C

SNP ID	rs174562	rs174564	rs28456	rs174566	rs174567	rs174568	rs99780	rs1535	rs174574	rs174576	rs174577	rs174578	rs174580	rs174581	
Modern human Haplotype D	A	A	A	A	A	C	C	A	C	C	C	T	A	G	
Modern human Haplotype A	G	G	G	G	G	T	T	G	A	A	A	A	G	A	risk for BD
Neanderthal	A	G	G	G	G	C	T	G	A	A	A	A	G	A	
Denisovan	G	G	G	G	T	T	G	A	A	A	A	A	G	G	
Chimpanzee	G	G	G	G	G	T	T	G	A	A	A	A	G	A	

Supplementary Fig. 1

The linkage disequilibrium (LD) block around the *FADS1* and *FADS2* genes.

a Regional association plot of the *FADS1/2* locus in BD according to GWASs (modified from [4]). The most significant SNP (rs174592) is shown by a purple diamond; the other SNPs are colored according to their degree of LD (r^2) with rs174592. The local LD structure is reflected by recombination rates (blue line). **b** Nucleotide sequences at the 28 SNP positions in the LD (modified from [4]). The majority of modern humans carry one of two haplotypes (D or A) within the LD block. Haplotype A, which is the ancestral haplotype shared among Hominini, confers risk for BD according to GWASs [1–3].

Supplementary Figure 2

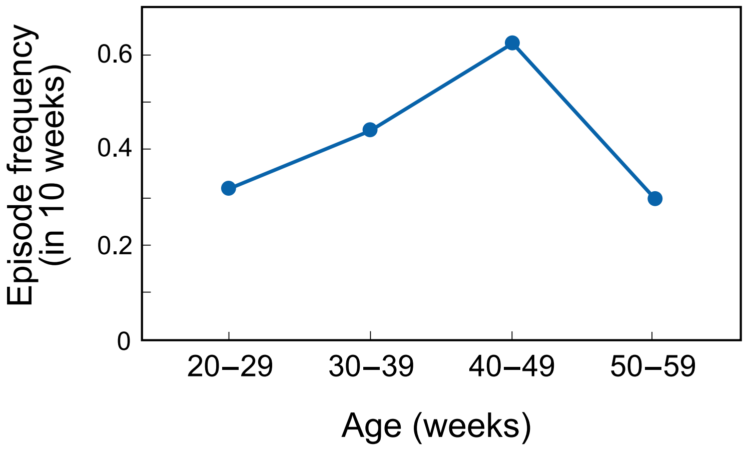

Supplementary Fig. 2

Age-dependent frequency of depressive-like episodes in female *Fads*(Δ/+) mice.

Episode frequency was calculated based on wheel-running activity data from 68 female *Fads*(Δ/+) mice fed a normal chow diet.

Supplementary Figure 3

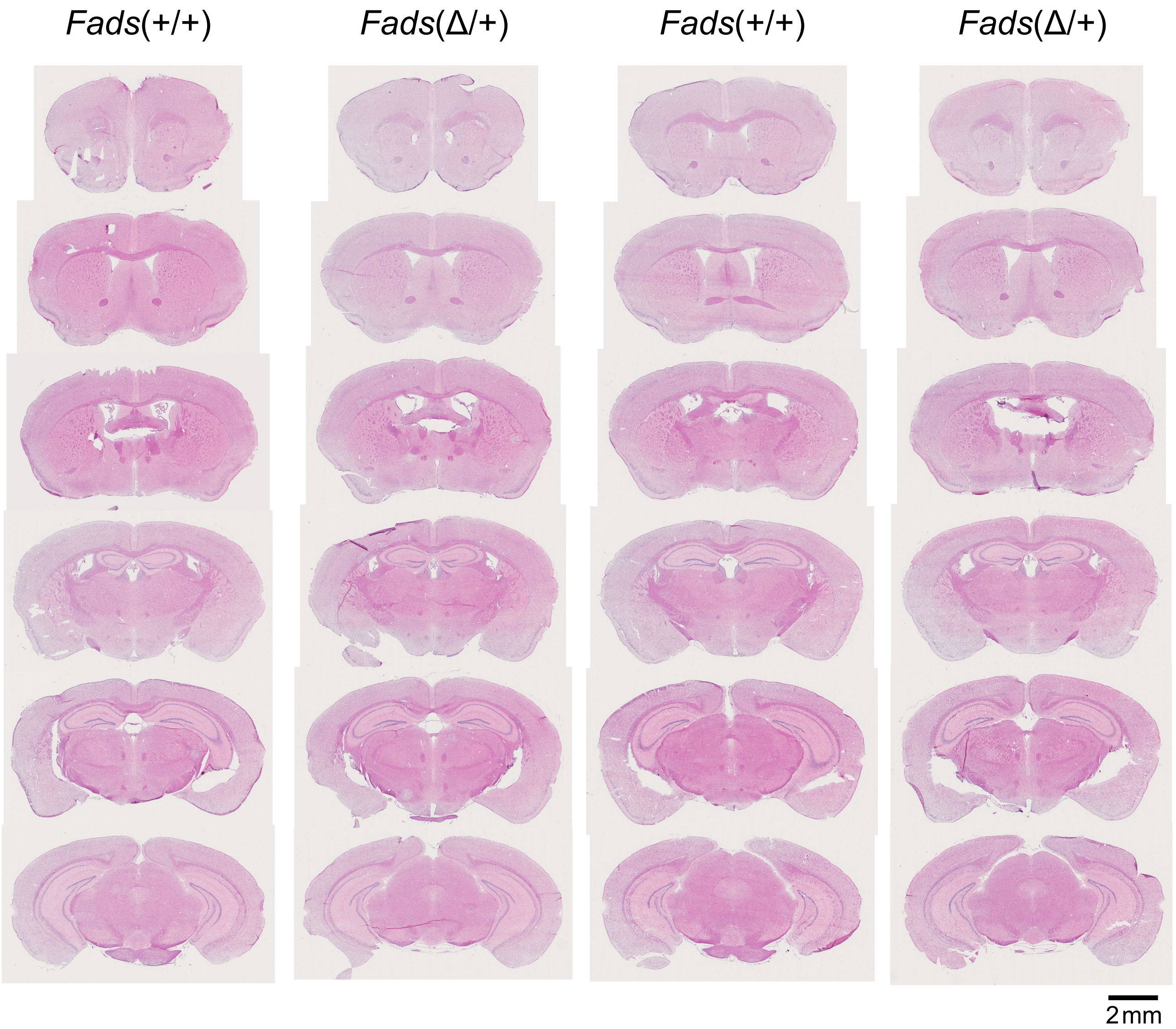

Supplementary Fig. 3

Hematoxylin-eosin (HE) staining of brains from *Fads*(Δ/+) mice and WT littermates.

Female mice (39 weeks old) were fixed with 4% paraformaldehyde. HE staining was performed on 10-μm sections. The stained sections were scanned with a digital image analyzer (Nanozoomer S60, Hamamatsu Photonics, Japan). There is no apparent difference in gross brain structure between *Fads*(Δ/+) and WT mice.

Supplementary Figure 4

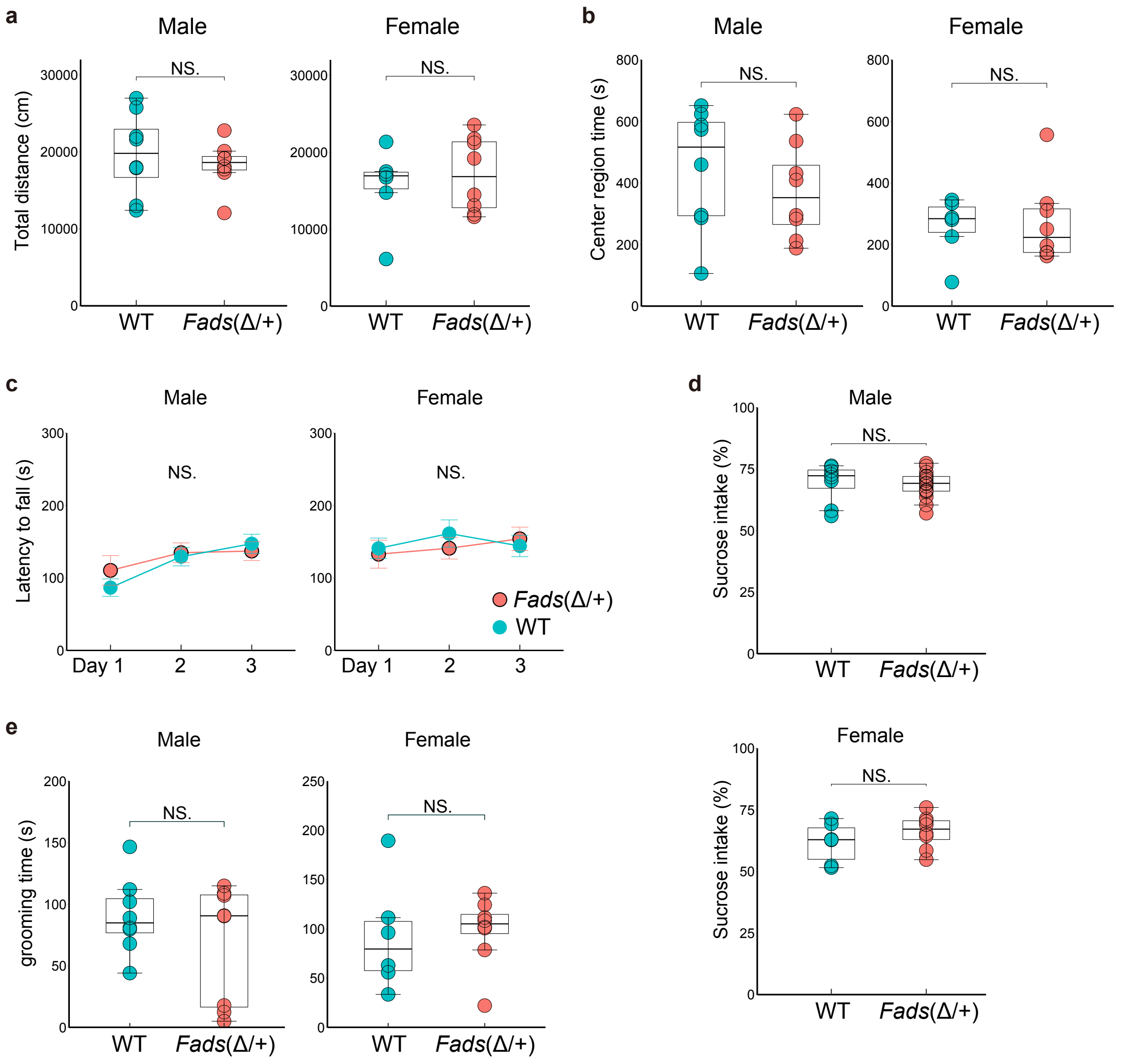

Supplementary Fig. 4

Results of conventional behavioral test battery of *Fads*(Δ/+) and WT mice carried out during the non-episodic state.

Fads(Δ/+) mice (20–35 weeks old at the start of the test battery) and WT littermates were used (male *Fads*(Δ/+), n = 8; male WT, n = 8; female *Fads*(Δ/+), n = 6; and female WT, n = 8). **a**, **b** Open-field test. There was no significant difference (NS) in either total distance traveled or time spent in the center region (t-test). For the boxplot description, see the legend of Fig. 1c. **c** Rotarod test. There was no significant difference in average latency to fall (two-way ANOVA). **d** Sucrose preference test. There was no significant difference in percentage of sucrose intake per total fluid intake (t-test). For this test only, the number of mice used was as follows: male *Fads*(Δ/+), n = 16; male WT, n = 8; female *Fads*(Δ/+), n = 8; and female WT, n = 6. **e** Splash test. There was no significant difference in grooming time (t-test).

Supplementary Figure 5

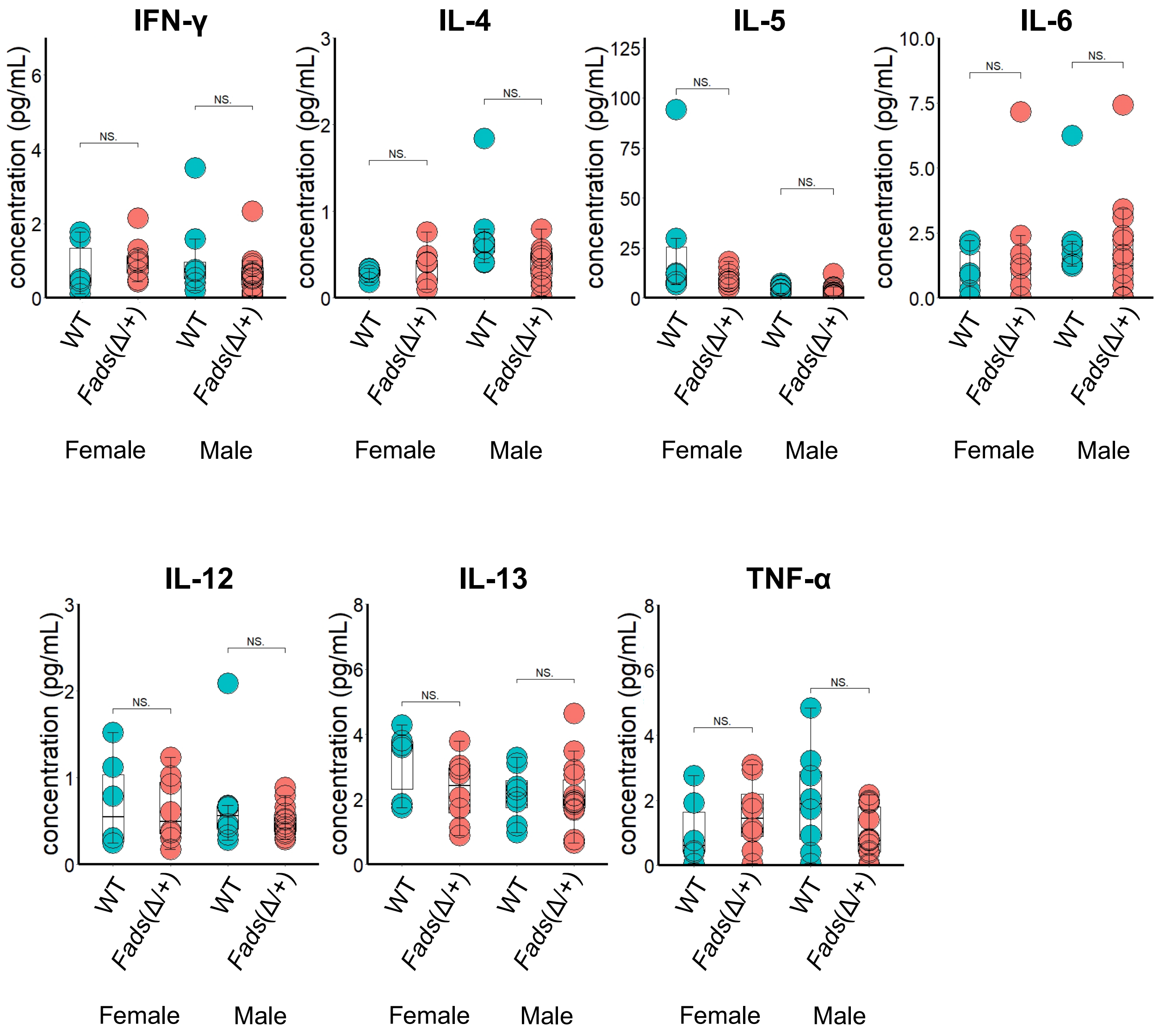

Supplementary Fig. 5

Plasma inflammatory markers of *Fads*(Δ/+) and WT mice fed a normal diet.

Eleven plasma inflammatory markers in *Fads*(Δ/+) and WT mice (23–38 week old; male *Fads*(Δ/+), *n* = 14; male WT, *n* = 8; female *Fads*(Δ/+), *n* = 8; and female WT, *n* = 6) were measured using ProcartaPlex Mouse Th1/Th2 Cytokine Panel 11prex (Thermo Fisher Scientific, MA, USA). Males and females were analyzed separately. Since more than 10% of the samples had values below the detection limit, 4 markers (GM-CSF, IL-1β, IL-2, and IL-18) were excluded from the statistical analysis. There was no significant difference in any plasma inflammatory markers between *Fads*(Δ/+) and WT mice during the non-episodic state. For the boxplot description, see the legend of Fig. 1c.

Supplementary Figure 6

a

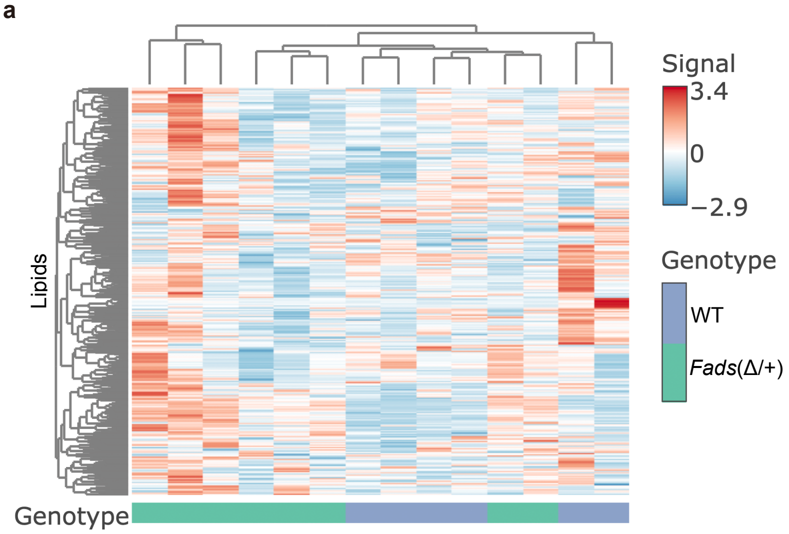

b

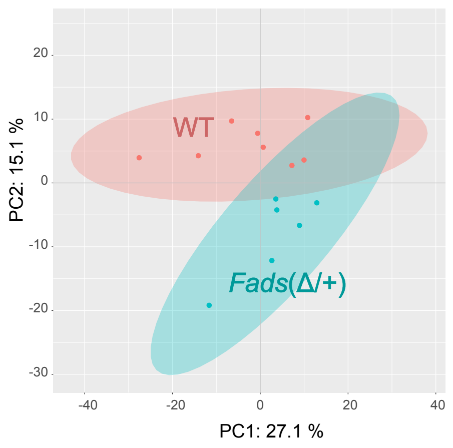

c

	Lipid class	Enrichment *P*-value	Effect size (*φ*)
Increased	None		
Decreased	None		

d

	Fatty acid	Enrichment *P*-value	Effect size (*φ*)
Increased	20:3	<0.0001	2.899 [large]
Decreased	None		

e

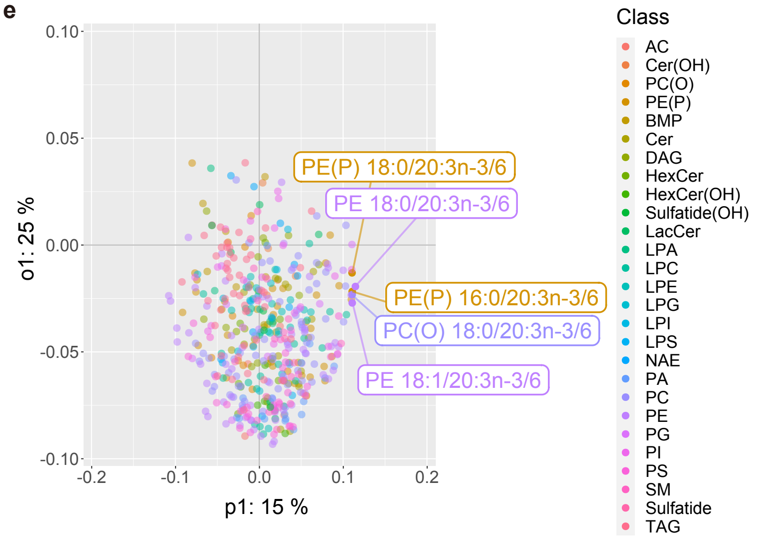

Supplementary Fig. 6

Brain lipid analysis of female *Fads*(Δ/+) and WT mice fed a normal chow diet.

a Dendrogram of unsupervised clustering analysis of 478 brain lipids in female mice fed normal chow. **b** PCA plot of female mice based on brain lipids. **c**,**d** Lipid class (**c**) and fatty acid (**d**) enrichment analysis of differentially changed brain lipids between *Fads*(Δ/+) and WT mice. Enrichment *P*-values are given by Fisher's exact test. The abbreviations for the lipid classes are listed in Supplementary Table 4. **e** OPLS-DA loading plot of the 478 brain lipids from *Fads*(Δ/+) and WT mice. The top 1% of lipid molecules are highlighted.

Supplementary Figure 7

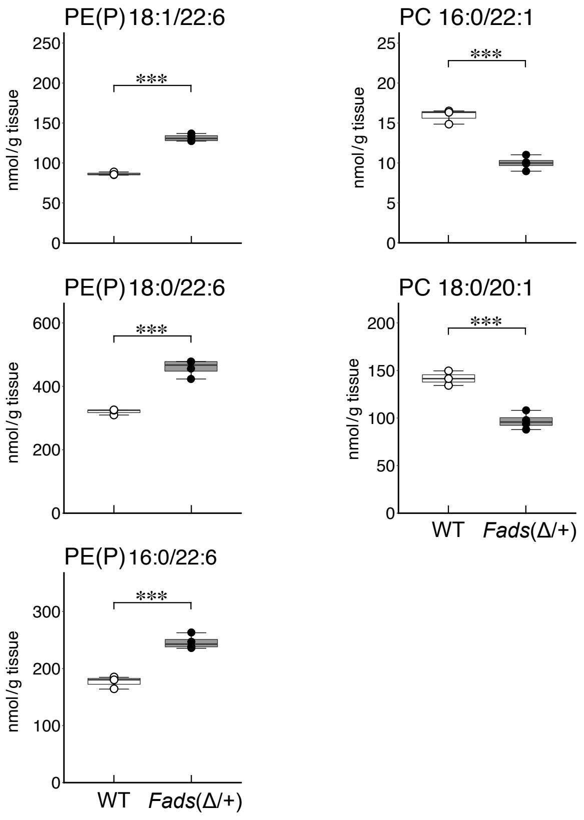

Supplementary Fig. 7

The top 1% of brain lipids between male WT and *Fads*(Δ/+) mice fed normal chow identified by an orthogonal partial least squares discriminant analysis (OPLS-DA).

Comparisons between the genotypes for the 5 lipids were performed using a two-tailed *t*-test (***$P < 0.001$, $d > 2.97$ [large ES]). Curiously, all the lipids that were increased in *Fads*(Δ/+) mice contained DHA (22:6). Without the a priori information given by OPLS-DA (Fig. 2f), only PE(P) 18:1/22:6 was significantly different between genotypes at FDR < 0.05. For the boxplot description, see the legend of Fig. 1c.

Supplementary Figure 8

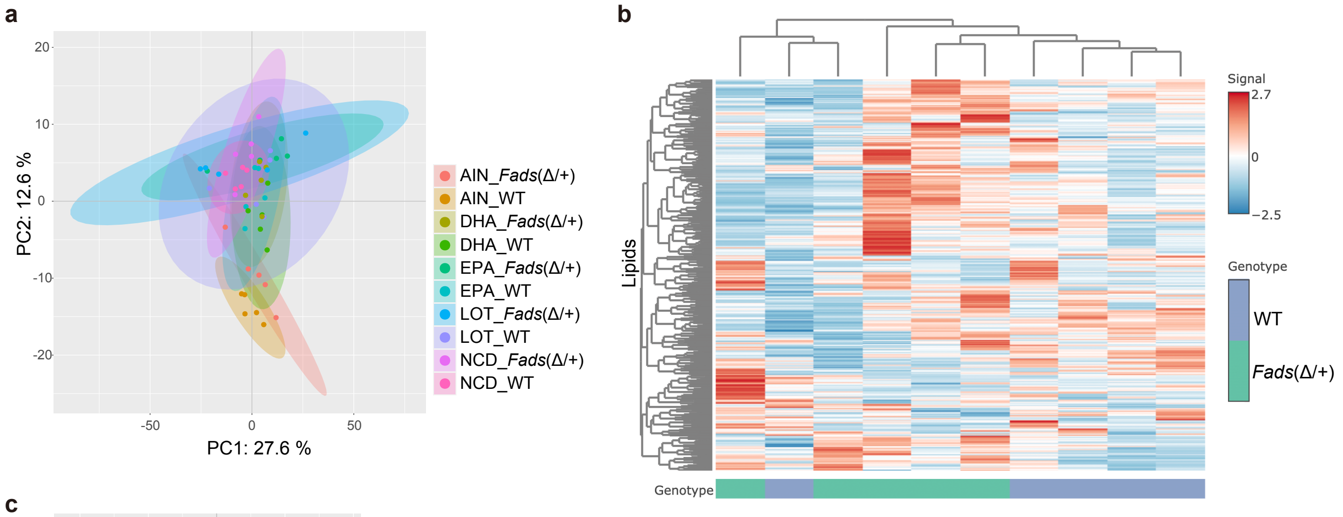

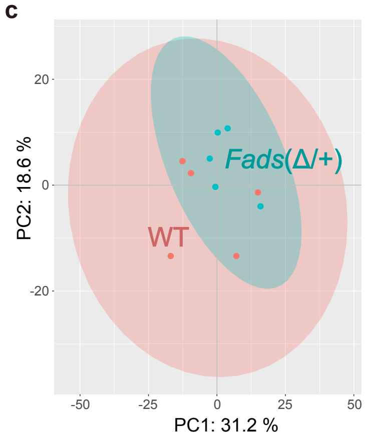

d

	Lipid class	Enrichment *P*-value	Effect size (*φ*)
Increased	PC	0.0087	0.424 [small]
Decreased	LPE	0.0024	1.157 [large]

e

	Fatty acid	Enrichment *P*-value	Effect size (*φ*)
Increased	20:2	<0.0001	2.442 [large]
	20:1	0.0203	0.309 [small]
Decreased	20:5	0.0330	0.511 [medium]

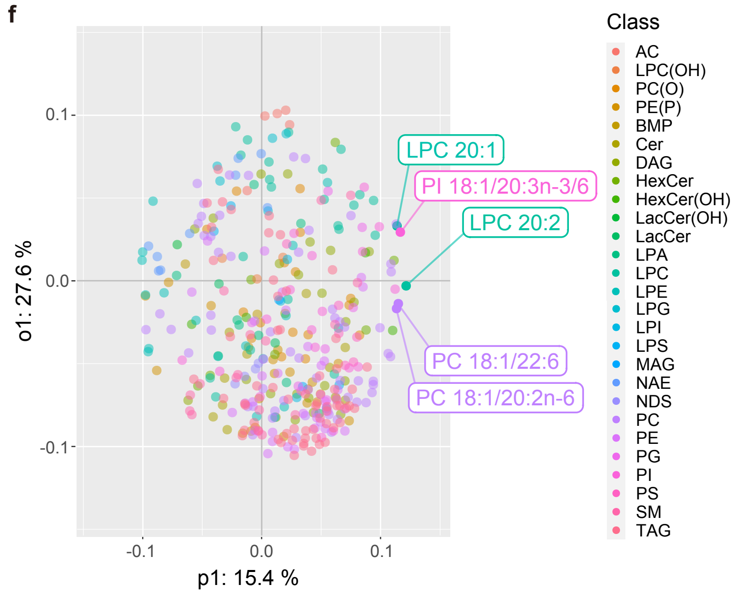

Supplementary Fig. 8

Plasma lipid analysis of male *Fads*(Δ/+) and WT mice fed a normal chow diet.

a PCA plot of *Fads*(Δ/+) and WT male mice fed five different diets based on plasma lipidomics data (Supplementary Table 1a). A two-way ANOVA revealed a significant and large effect of genotype and diet ($P < 0.001$) on the Euclidean distance between the lipid compositions of the samples measured. AIN, AIN93G diet; DHA, AIN93G+DHA; EPA, AIN93G+EPA; LOT, AIN93G+EPA+DHA; NCD, normal chow diet. **b** Dendrogram of unsupervised clustering analysis of 383 plasma lipids in male mice fed a normal chow diet. **c** PCA plot of male mice based on plasma lipids. **d**,**e** Lipid class (**d**) and fatty acid (**e**) enrichment analyses of differentially changed plasma lipids between *Fads*(Δ/+) and WT mice fed normal diet. The abbreviations for the lipid classes are listed in Supplementary Table 4. **f** OPLS-DA loading plot of the 385 plasma lipids from *Fads*(Δ/+) and WT mice fed a normal chow diet. The top 1% of lipid molecules are highlighted.

Supplementary Figure 9

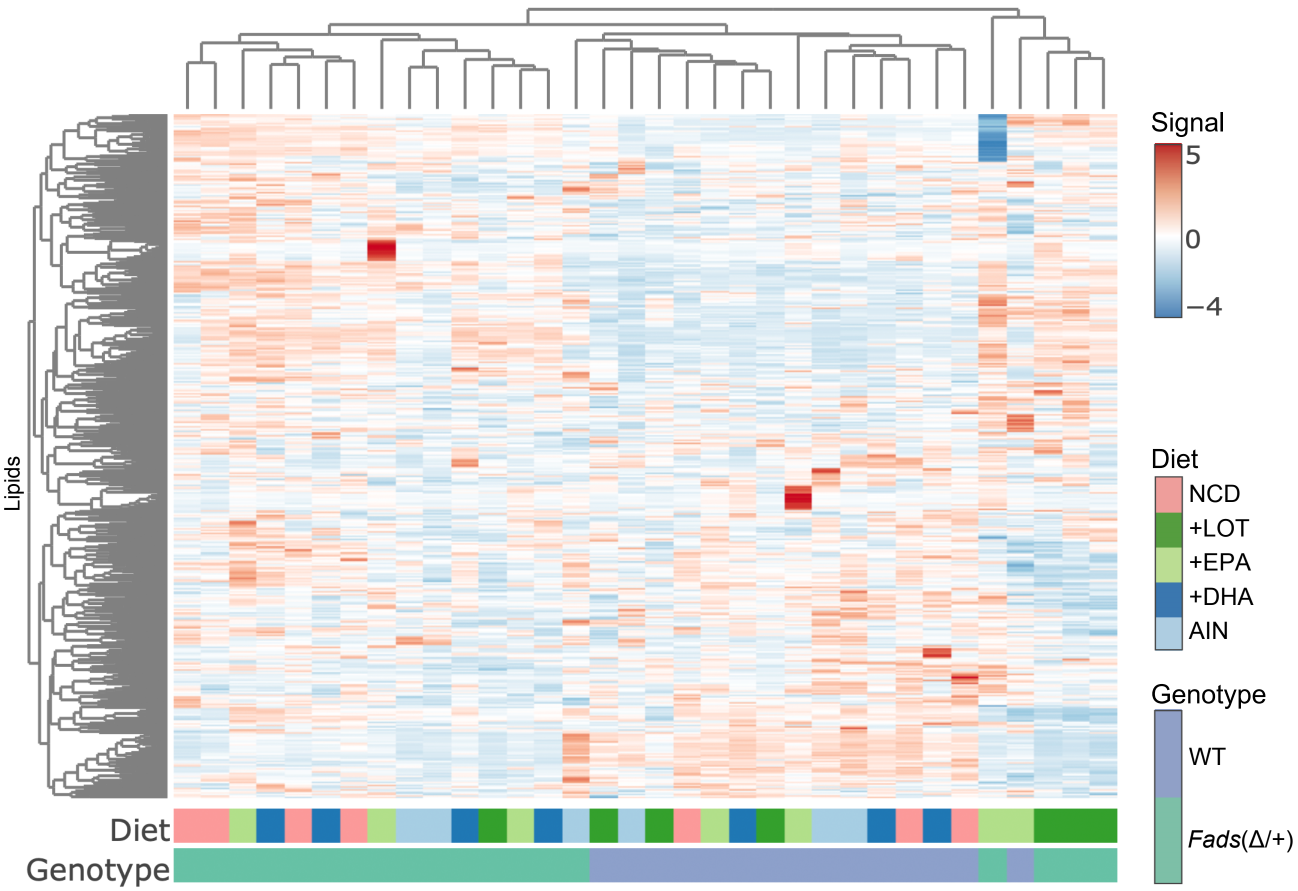

Supplementary Fig. 9

Lipidomics of brain tissues in *Fads*(Δ/+) and WT mice fed five different diets.

Dendrogram of unsupervised clustering analysis of 464 lipids of *Fads*(Δ/+) and WT mice fed five differnet diets. AIN, AIN93G diet; DHA, AIN93G+DHA; EPA, AIN93G+EPA; LOT, AIN93G+EPA+ DHA; NCD, normal chow diet.

Supplementary Figure 10

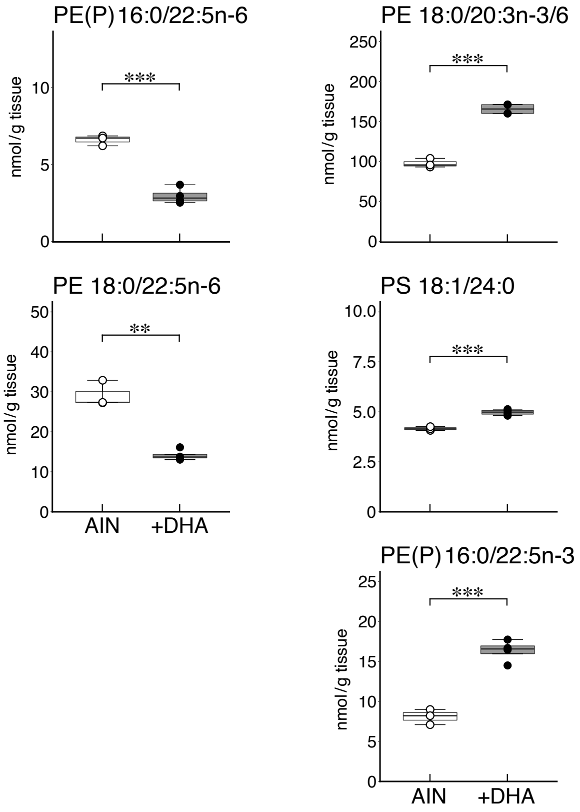

Supplementary Fig. 10

The top 1% of brain lipids between mice fed AIN93G and those supplemented with DHA identified by OPLS-DA.

Comparisons between the diets for the 5 lipids were performed using a two-tailed *t*-test (***P* < 0.01, ****P* < 0.001, *d* = 0.569 [medium ES] for PS 18:1/24:0, and *d* > 2.24 [large ES] for others). Without the a priori information provided by OPLS-DA (Fig. 4e), all 5 lipids were significantly different at FDR < 0.05. For the boxplot description, see the legend of Fig. 1c.

Supplementary Figure 11

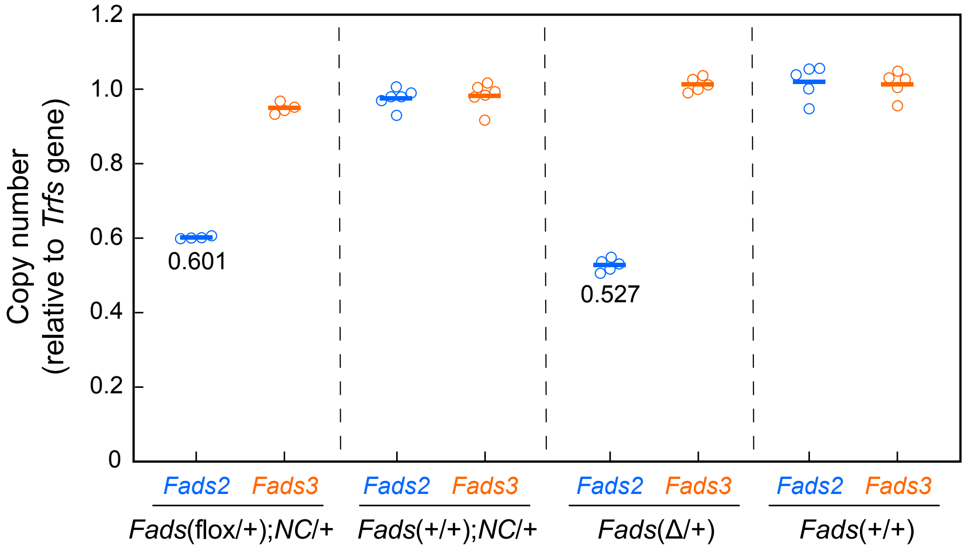

Supplementary Fig. 11

Digital PCR (dPCR) analysis of the genomes of brains in *Fads1/2* mutant mice.

DNA was extracted from a whole mouse brain of each genotype and the copy numbers of the *Fads2* and *Fads3* regions were quantified by dPCR. The *Trfs* region present in another autosome was used as a reference (2 copies per diploid) to show copy numbers of *Fads2* and *Fads3* regions. The *Fads3* region was not deleted in any genotype, as expected (Fig. 1b); the *Fads2* region was deleted in ~80% of cells in heterozygous cKO mice [*Fads*(flox/+);*NC*/+] and in ~95% of cells in heterozygous KO mice [*Fads*(Δ/+)].

Supplementary Figure 12

a

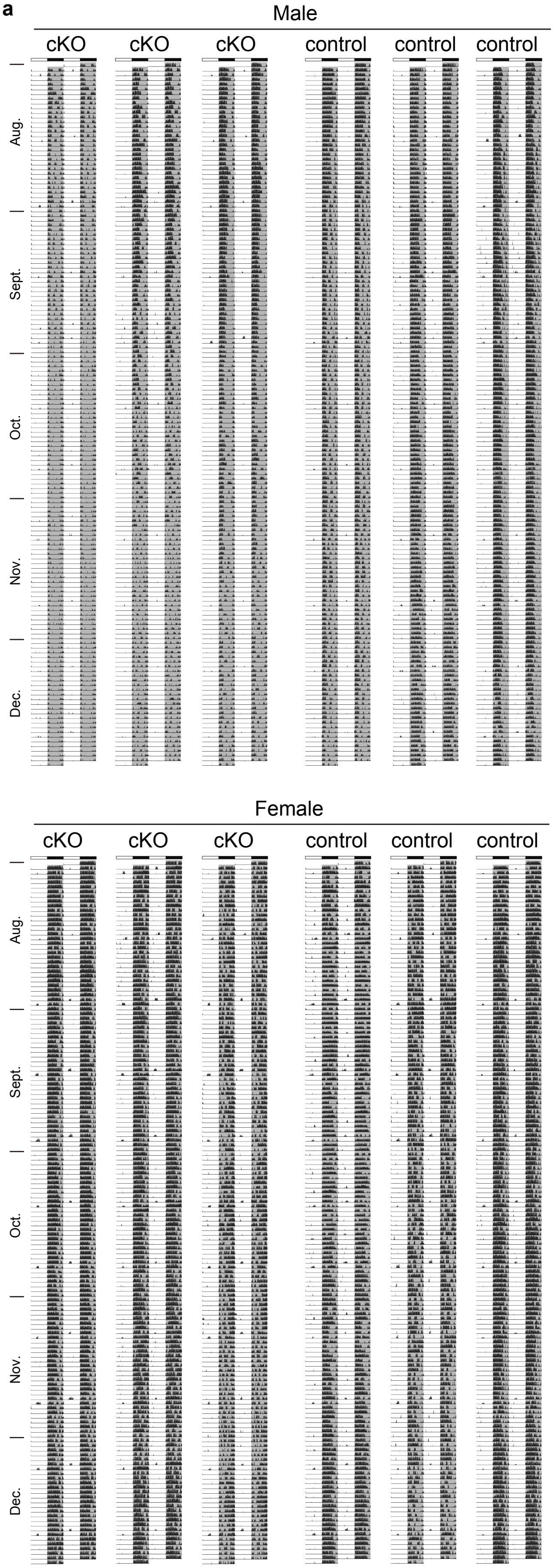

b

Sex	Genotype	*n*	N of individuals exhibiting HABs	Average N of HABs (per 6 months)
Male	*Fads*(flox/+);*NC*/+	20	0	0
	Fads(+/+);*NC*/+	17	1	0.06
Female	*Fads*(flox/+);*NC*/+	11	0	0
	Fads(+/+);*NC*/+	10	0	0

c

Sex	Genotype	*n*	N of individuals exhibiting DEs	Average N of DEs (per 6 months)
Male	*Fads*(flox/+);*NC*/+	20	0	0
	Fads(+/+);*NC*/+	17	0	0
Female	*Fads*(flox/+);*NC*/+	11	0	0
	Fads(+/+);*NC*/+	10	0	0

Supplementary Fig. 12

Brain-specific deletion of the *Fads1/2* genes had no significant impact on episodic behavioral phenotypes.

a Representative double-plotted actograms of wheel-running activity in brain-specific *Fads1/2* KO mice [*Fads*(flox/+);NC/+] (cKO) and control mice [*Fads*(+/+);NC/+] fed normal chow diet.

b Frequency of HABs. Neither male nor female mice showed any statistical differences.

c Frequency of depression-like episodes (DEs). No episodic behavioral changes were observed.

Supplementary Figure 13

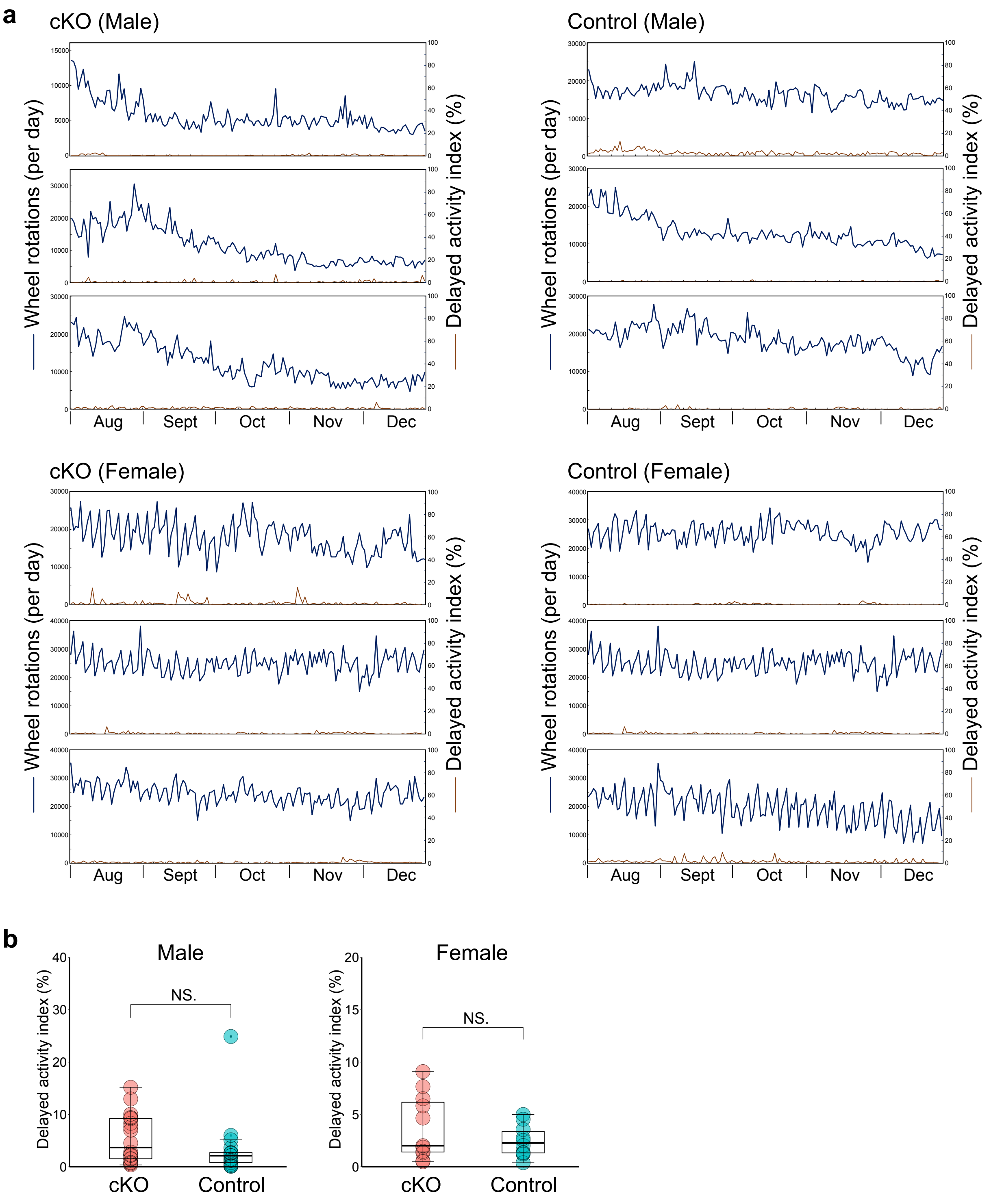

Supplementary Fig. 13

Brain-specific deletion of the *Fads1/2* genes had no significant impact on daily wheel-running activity or sleep-wake rhythm.

a Representative recording of daily wheel rotations and delayed activity index of brain-specific *Fads1/2* KO mice (cKO) or control mice. Individual data are identical to those actograms in Supplementary Fig. 12. **b** Average delayed activity index. The averages of delayed activity index for male (cKO, n = 20; Control, n =17) and female (cKO, n = 11; Control, n =10) mice for approximately 5 months are shown, respectively. There was no significant difference in delayed activity index (t-test). For the boxplot description, see the legend of Fig. 1c.

Supplementary Figure 14

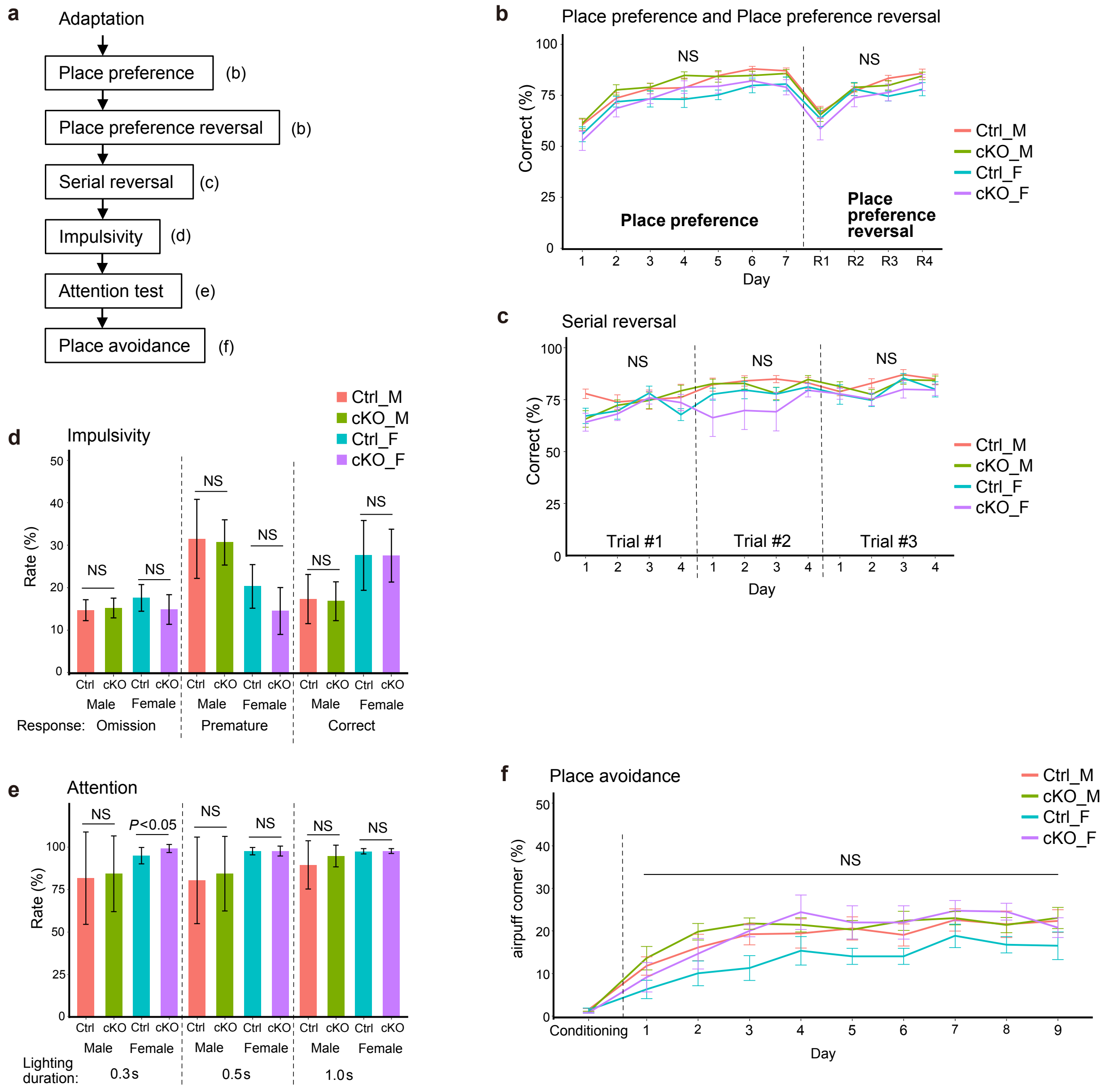

Supplementary Fig. 14

***Fads* cKO mice had no apparent phenotype of non-episodic behaviors in the IntelliCage.**

a Schedule of non-episodic behavioral tests using IntelliCage. **b** Percentage of correct corners visited during the place preference and place preference reversal tests. A repeated-measures ANOVA revealed no significant difference between *Fads*(+/+);*NC*/+ (Ctrl) and *Fads*(flox/+);*NC*/+ (cKO) mice, for either males or females. Male Ctrl, *n* = 11; male cKO, *n* = 12; female Ctrl, *n* = 6; female cKO, *n* = 8. **c** Percentage of correct corners visited during the serial reversal. A repeated-measures ANOVA revealed no significant difference between Ctrl and cKO mice. **d** Nose poke percentages in the impulsivity test. There was no significant difference in impulsive behavior between the genotypes. **e** Nose poke percentages in the attention test. There was no significant difference in attention behavior between the genotypes. **f** Percentage of visiting airpuff corners during the place avoidance test. Airpuff conditioning worked well for each mouse group. A repeated-measures ANOVA revealed no significant difference between Ctrl and cKO mice for either males or females.

Supplementary Figure 15

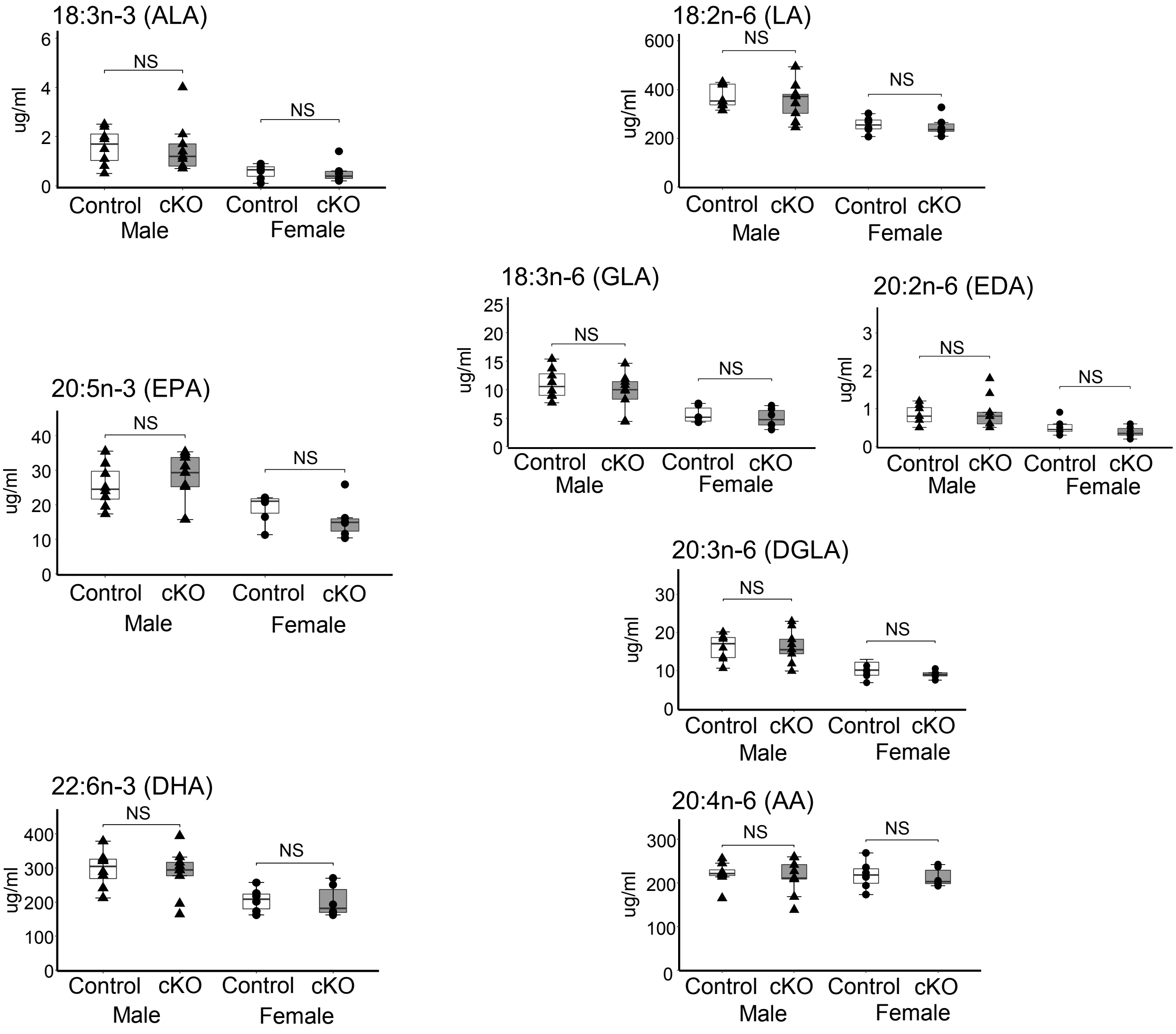

Supplementary Fig. 15

Plasma fatty acids levels in brain-specific Fads1/2 KO mice.

Plasma fatty acid levels in *Fads*(flox/+);*NC*/+ (cKO) and *Fads*(+/+);*NC*/+ (control) mice fed normal chow. 18:3n-6 (GLA) and 20:2n-6 (EDA), which were significantly changed in *Fads*(Δ/+) mice (Fig. 2a), did not change in cKO mice (male, *n* = 9; female, *n* = 6) *vs.* control mice (male, *n* = 8; female, *n* = 6). For the boxplot description, see the legend of Fig. 1c.

Supplementary Figure 16

a

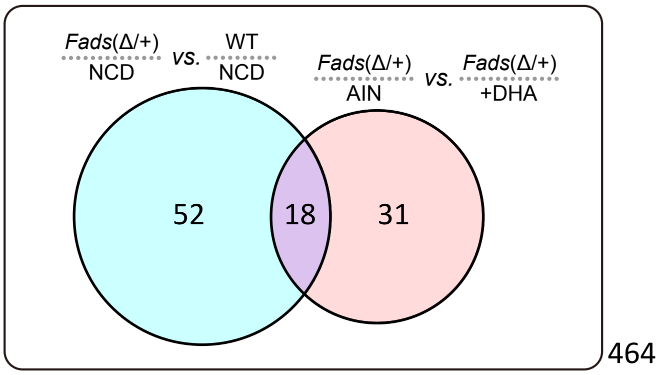

b

Lipids	Fold change (*Fads*(Δ/+)/WT)	*P*-value	Effect size (*d*)	Fold change (AIN/DHA)	*P*-value	Effect size (*d*)
DAG 20:5/18:0	3.1980	0.0077	1.5710	0.3258	0.0059	1.8960
PE 16:0/20:3n-3/6	2.4118	0.0228	1.2730	0.5595	0.0010	3.0630
PE 18:0/20:3n-3/6	2.3137	0.0421	1.0750	0.5885	0.0000	6.5720
PE 18:1/20:3n-3/6	2.2611	0.0183	1.3780	0.6408	0.0061	1.9270
PE(P) 18:1/20:3n-3/6	2.0325	0.0335	1.1520	0.6647	0.0005	3.9910
PS 18:0/20:3	1.7737	0.0088	1.6590	0.7182	0.0024	3.1570
PS 18:1/20:3	1.7627	0.0031	1.3240	0.7811	0.0120	0.8210
PE 18:2/18:1	1.7115	0.0146	1.4500	0.7431	0.0415	1.3570
PE 22:6/22:6	1.5649	0.0188	1.3870	0.6856	0.0335	1.2890
PE(P) 18:1/22:5n-3	1.5315	0.0362	1.0700	0.4716	0.0008	3.8130
PE(P) 18:0/22:6	1.4336	0.0003	3.5200	0.8086	0.0468	1.5080
PE(P) 16:0/22:6	1.3936	0.0006	3.5140	0.7771	0.0308	1.1940
PE 18:0/22:6	1.3694	0.0082	1.9860	0.8596	0.0213	1.8290
PS 18:1/18:1	1.2962	0.0007	3.3360	0.8841	0.0066	2.4140
PE 18:1/18:1	1.2867	0.0204	1.4600	0.7575	0.0314	1.2830
PS 18:1/20:1	1.2792	0.0018	1.8560	0.8973	0.0272	0.9030
PE(P) 18:1/18:1	1.1805	0.0106	2.0040	0.8392	0.0282	1.3150
PS 18:1/20:0	1.1416	0.0020	1.4420	0.9059	0.0412	0.9130

Supplementary Fig. 16

Brain lipids that changed in comparison between male *Fads*(Δ/+) mice fed a normal chow and WT controls and that changed in comparison between male *Fads*(Δ/+) mice fed AIN93G diet and male *Fads*(Δ/+) mice fed DHA-supplemented AIN93G diet.

a Among 464 lipids measured using LC-MS/MS, 70 lipids were significantly ($P < 0.05$ and $d > 0.8$) changed in comparison between *Fads*(Δ/+) mice fed a normal chow (NCD) and WT controls fed NCD and 49 lipids were in comparison between male *Fads*(Δ/+) mice fed AIN93G diet (AIN) and male *Fads*(Δ/+) mice fed DHA-supplemented AIN93G diet (+DHA). 18 lipids were common between the comparisons, and Monte Carlo simulations showed that significantly more lipids were shared ($P < 0.001$). **b** List of the shared 18 lipids. The directions of changes in the lipid levels are in conflict with the simple assumption that the DHA-supplemented diet would alter the brain lipid composition of *Fads*(Δ/+) mice to that of WT mice. The probability that the directions of changes in all those lipids are opposite in the two comparisons is $P < 0.0001$.
